# The effectiveness of training strategies to improve healthcare provider practices in low-income and middle-income countries

**DOI:** 10.1136/bmjgh-2020-003229

**Published:** 2021-01-15

**Authors:** Alexander K Rowe, Samantha Y Rowe, David H Peters, Kathleen A Holloway, Dennis Ross-Degnan

**Affiliations:** 1 Division of Parasitic Diseases and Malaria, Centers for Disease Control and Prevention, Atlanta, Georgia, USA; 2 CDC Foundation, Atlanta, Georgia, USA; 3 Department of International Health, Johns Hopkins Bloomberg School of Public Health, Baltimore, Maryland, USA; 4 International Institute of Health Management Research, Jaipur, India; 5 Institute of Development Studies, University of Sussex, Brighton, Brighton and Hove, UK; 6 Department of Population Medicine, Harvard Medical School and Harvard Pilgrim Health Care Institute, Boston, Massachusetts, USA

**Keywords:** health services research

## Abstract

**Introduction:**

In low/middle-income countries (LMICs), training is often used to improve healthcare provider (HCP) performance. However, important questions remain about how well training works and the best ways to design training strategies. The objective of this study is to characterise the effectiveness of training strategies to improve HCP practices in LMICs and identify attributes associated with training effectiveness.

**Methods:**

We performed a secondary analysis of data from a systematic review on improving HCP performance. The review included controlled trials and interrupted time series, and outcomes measuring HCP practices (eg, percentage of patients correctly treated). Distributions of effect sizes (defined as percentage-point (%-point) changes) were described for each training strategy. To identify effective training attributes, we examined studies that directly compared training approaches and performed random-effects linear regression modelling.

**Results:**

We analysed data from 199 studies from 51 countries. For outcomes expressed as percentages, educational outreach visits (median effect size when compared with controls: 9.9 %-points; IQR: 4.3–20.6) tended to be somewhat more effective than in-service training (median: 7.3 %-points; IQR: 3.6–17.4), which seemed more effective than peer-to-peer training (4.0 %-points) and self-study (by 2.0–9.3 %-points). Mean effectiveness was greater (by 6.0–10.4 %-points) for training that incorporated clinical practice and training at HCPs’ work site. Attributes with little or no effect were: training with computers, interactive methods or over multiple sessions; training duration; number of educational methods; distance training; trainers with pedagogical training and topic complexity. For lay HCPs, in-service training had no measurable effect. Evidence quality for all findings was low.

**Conclusions:**

Although additional research is needed, by characterising the effectiveness of training strategies and identifying attributes of effective training, decision-makers in LMICs can improve how these strategies are selected and implemented.

**Key questions**

**What is already known?**

- In low/middle-income countries (LMICs), training is often used to improve healthcare provider (HCP) performance, however studies have found that training’s effect is highly variable.
- Thus, important questions remain about how to optimise training effectiveness.

**What are the new findings?**

- Although evidence quality for all findings was low, educational outreach visits tended to be somewhat more effective than in-service training, which seemed more effective than peer-to-peer training and self-study.
- Mean effectiveness was greater for training that incorporated clinical practice and training at HCPs’ work site.
- For lay HCPs (community health workers), in-service training had no measurable effect.

**What do the new findings imply?**

- Although additional research is needed, for now, decision-makers in LMICs might improve training effectiveness by selecting the approaches identified in this study.

## Introduction

Healthcare providers (HCPs), including facility-based and community-based health workers, play essential roles in delivering healthcare. However, hundreds of studies have documented inadequate HCP performance in low/middle-income countries (LMICs).^1 2^ Estimates of the consequences of poor-quality care range between 4.9–5.2 million^2^ and 5.7–8.4 million^3^ deaths annually.

Strategies to train HCPs are often used to improve performance. For example, from 1988 to 1993, to promote oral rehydration therapy, more than 2000 training courses were held on managing diarrhoea in more than 120 countries.^4^ From 2011 to 2015, US$95.3 million in country proposals were approved by Gavi for health workforce development and, in particular, training and capacity building for HCPs in LMICs (personal communication, Alan Brooks, Gavi, 17 November 2015). From 2017 to 2019, all 72 country-specific annual plans of the President’s Malaria Initiative funded training.^5–7^ For the 2018–2020 funding cycle, grant recipients of The Global Fund to Fight AIDS, Tuberculosis and Malaria (GFATM) have budgeted US$510 million for training (personal communication, Benjamin Loevinsohn, GFATM, 15 December 2019).

Despite widespread use, important questions remain about training effectiveness and the best ways to design training strategies in LMICs. A large systematic review of studies from LMICs (the Health Care Provider Performance Review (HCPPR)) found that training was the most often studied strategy, and its effect on HCP practices varied substantially, from –19.9 to 60.8 percentage-points (%-points) (median: 10.3, IQR: 6.1–20.7).^1^ However, that analysis combined multiple strategies (eg, group training, self-study and peer-to-peer training) and implementation approaches (eg, in terms of educational methods and training duration) into a single heterogeneous group. Other reviews that examined training effectiveness or sought to identify best practices for designing training courses have key limitations, such as providing only non-quantitative, narrative summaries (which obscures the magnitude of effect of recommended design attributes), or including few studies from LMICs.^8–12^

This report presents a secondary analysis of HCPPR data to characterise the effectiveness of different training strategies (objective 1) and identify attributes associated with group in-service training effectiveness (and thus suggest ways to increase training effectiveness) (objective 2). The results can inform the choice and design of future training strategies and shape the research agenda on improving training effectiveness.

## Methods

This report uses the same methods as those used in an HCPPR-based analysis of supervision strategies.^13^ We analysed data from the HCPPR (PROSPERO registration CRD42016046154). Details of the HCPPR’s inclusion criteria, literature search, data abstraction, risk of bias assessment, effect size estimation and assessment of publication bias have been published elsewhere.^1 14^ A summary of those methods and additional details particular to this report are presented below and in online supplemental appendix 1 (section 1).

10.1136/bmjgh-2020-003229.supp1 Supplementary data

### Inclusion criteria

The HCPPR included published and unpublished studies from LMICs in the public and private sectors that quantitatively evaluated a strategy to improve HCP performance. HCPs were broadly defined as hospital-based, health facility-based or community-based health workers; pharmacists and shopkeepers who sell medicines. Eligible study designs included pre-intervention versus post-intervention studies with a randomised or non-randomised comparison group, post-intervention only studies with a randomised comparison group and interrupted time series (ITS). ITS studies could have a randomised or non-randomised control group, or no separate control group (see online supplemental appendix 1, table B1). The HCPPR included studies on any health condition and there were no language restrictions. For outcomes expressed as a percentage, effect sizes with baseline values of 95% or greater were excluded, as there was little room for improvement.

For this report, we only included studies that tested strategies with a component to train HCPs, although many strategies also had other intervention components. Additionally, we only analysed HCP practice outcomes (eg, patient assessment, diagnosis, treatment, counselling, referral, documentation and consultation time). This outcome category had the largest number of studies in the HCPPR.

### Literature search and data abstraction

The HCPPR searched 52 electronic databases for published studies and 58 document inventories for unpublished studies from the 1960s to 2016. Personal libraries were screened, experts were queried for unpublished studies and hand searches were performed of bibliographies from previous reviews.

To identify eligible reports, titles and abstracts were screened, and when necessary, a report’s full text was reviewed. Data were abstracted independently by two investigators or research assistants using a standardised form. Discrepancies were resolved through discussion. Data were entered into a database (Microsoft Access, Microsoft, Redmond, Washington, USA). Data elements included HCP type, improvement strategies, outcomes, effect sizes and risk of bias domains. Study investigators were queried about details not available in study reports. The data and an associated data dictionary are publicly available at http://www.HCPperformancereview.org.

### Strategy definitions

In the HCPPR database, the presence of 207 strategy components was coded for each study arm exposed to an improvement strategy, and the components were grouped into categories. For HCP training strategies, five specific categories were created (eg, group in-service training) (box 1, top part). Eleven other categories were more general (eg, supervision, group problem-solving). Placebo strategy components were analysed together with control groups that received no new intervention.

Box 1 **Strategy definitions**

**Training strategies for healthcare providers (HCPs) (all categories are mutually exclusive)**

1. Group in-service training. On-the-job training primarily led by a facilitator, typically for a group of HCPs in a classroom or clinical setting.*
2. Group preservice training. Training for HCPs before they begin practising, primarily led by a facilitator, typically for a group of HCPs in a classroom or clinical setting.*
3. Self-study in-service training. On-the-job training with structured sessions in which HCPs study by themselves without direct supervision or attendance in a class. HCPs might occasionally interact with a facilitator, supervisor or peer to discuss the training content.†
4. Educational outreach visits. On-the-job training strategy with face-to-face visits to individual HCPs at their workplace by persons who HCPs regard as an expert or opinion leader to promote best practices. Also known as academic detailing.
5. Peer-to-peer training. On-the-job training led by HCP peers usually at the HCPs’ workplace. For example, an HCP (eg, a facility in-charge) attends a training course and then returns to his/her clinic and shares the training information with other HCPs. Also known as peer education. This strategy is different from educational outreach visits because peer-to-peer training does not involve visits by an external expert or opinion leader.

**Other strategy components (all categories are mutually exclusive)‡**

1. Community support, for example, community health education or social marketing of health services.
2. Patient support, for example, patient health education via printed materials or home visits.
3. Strengthening infrastructure, for example, provision of medicines or equipment.
4. HCP-directed financial incentives, for example, performance-based payments.
5. Health system financing and other incentives, for example, insurance or reducing a consultation fee.
6. Regulation and governance, for example, accreditation system.
7. Group problem-solving, for example, collaborative improvement or group problem-solving with or without formal teams.
8. Supervision, for example, improving routine supervision, benchmarking or audit with feedback.
9. Other management techniques that do not include group problem-solving and supervision, for example, HCP self-assessment or HCP group process that is not group problem-solving.
10. Printed information or job aid for HCPs that is not an integral part of another component, for example, pamphlet for HCP.§
11. Information and communication technology (includes mHealth and eHealth) for HCPs, for example, computerised decision aid or text message reminders sent to HCPs’ phones.

*Trainees might spend some time working individually to learn the material (eg, reading to prepare for the next day’s class), however such activity was not considered self-study.†Does not include: (1) educational materials given to HCPs before group in-service training that HCPs are asked to review to prepare for that training (such materials are considered an integral part of the group training, rather than self-study), or (2) educational materials given to HCPs without specific instructions on how HCPs should learn them (eg, distributing a printed treatment manual with the instruction that HCPs should use it, but without further guidance).‡Detailed definitions in Appendix 1 of Rowe *et al*.^1^§Other strategy components (especially training) often include printed information for HCPs; and in these cases, the printed information was not considered a separate component.

### Risk of bias assessment

Risk of bias at the study level was categorised as low, moderate, high or very high. Randomised studies, ITS, and non-randomised studies were initially categorised as low, moderate and high risk of bias, respectively. Risk of bias domains (eg, dataset completeness, balance in baseline outcome measurements and so on) were then assessed. A study’s risk of bias category was dropped by one level for every applicable domain that was ‘not done’ and for every two applicable domains that were ‘unclear’.

### Estimating effect sizes

The primary outcome measure was the effect size, which was defined as an absolute %-point change and calculated such that positive values indicate improvement. For study outcomes that decreased to indicate improvement (eg, percentage of patients receiving unnecessary treatments), we multiplied effect sizes by –1. For non-ITS studies, effect sizes for outcomes expressed as a percentage (eg, percentage of patients treated correctly) were calculated with equation 1 (if there were baseline values) or equation 2 (if no baseline values). Effect sizes were based on the baseline value closest in time to the beginning of the strategy and the follow-up value furthest in time from the beginning of the strategy.


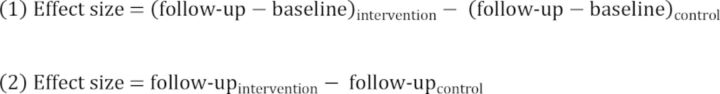


In non-ITS studies, effect sizes for unbounded continuous outcomes (eg, number of medicines prescribed per patient) were calculated with equation 3 (if there were baseline values) or equation 4 (if no baseline values). For unbounded continuous outcomes, if the baseline value for either the intervention or control group equaled zero (when equation 3 was used) or if the follow-up value for the control group equaled zero (when equation 4 was used), the effect size was undefined and thus excluded. Note that such exclusions were rare (<2%).


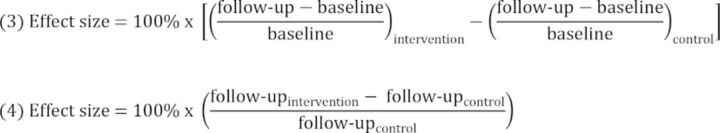


For ITS studies, segmented linear regression modelling was performed to estimate a summary effect size that incorporated both level and trend effects. The summary effect size was the outcome level at the midpoint of the follow-up period as predicted by the regression model minus a counterfactual value that equaled the outcome level based on the pre-intervention trend extended to the midpoint of the follow-up period.

### Analysis

- For objective 1 (characterise the effectiveness of training strategies), we analysed five types of study comparisons (box 2). To estimate strategy effectiveness, the effect size for each study comparison was defined as the median of all effect sizes (MES) within the comparison. That is, results of multiple outcomes from the same study comparison were collapsed into a single number. For example, if a study had three outcomes (eg, percentages of patients correctly assessed, diagnosed and treated) and one effect size per outcome, the MES was the median of the three effect sizes. For each training strategy, the MES distribution was described with a median, IQR, minimum and maximum. Results were stratified by outcome scale (percentage vs continuous), HCP cadre (professional (generally, facility-based health workers) vs lay (generally, community health workers)), whether the training strategy was combined with other intervention components, and study type (equivalency vs non-equivalency).

Box 2 **Study comparisons used to characterise the effectiveness of different training strategies (objective 1)**

**Non-equivalency studies (success is an effect size with a large magnitude)**

- Comparison of a training strategy* alone† versus a (no-intervention) control group.
- Comparison of one training strategy* alone† versus a different training strategy* alone.†
- Comparison of a training strategy* combined with a specific group of other strategy components versus that same specific group of other strategy components‡ (eg, ‘educational outreach visits plus supervision’ vs ‘supervision’).
- Comparison of one training strategy* combined with a specific group of other strategy components‡ versus a different training strategy* combined with that same specific group of other strategy components‡ (eg, ‘educational outreach visits plus supervision’ vs ‘group in-service training plus supervision’).

**Equivalency studies (success is an effect size close to zero)**

- Comparison of a training strategy* alone† versus a ‘gold standard’ comparison group (eg, intervention group of nurse-midwives trained to perform tubal ligation surgery vs a gold standard comparison group of physicians).

*Any of the five training strategies listed in the top part of box 1.†That is, not combined with other strategy components. Printed materials, which are usually used in training courses, were not considered to be a separate component (eg, the strategy ‘in-service training plus printed guideline’ was considered to be ‘in-service training alone’).‡One or more of the 11 strategy categories in the bottom part of box 1.

For objective 2 (identify attributes associated with group in-service training effectiveness), we used two approaches. First, we examined head-to-head studies that directly compared different training approaches (eg, 6-day vs 11-day training). As with objective 1, results of multiple outcomes from the same study comparison were collapsed into a single MES value. Second, we used random-effects linear regression modelling on studies of training with different approaches versus a control group. The dependent variable was the outcome-specific effect size (ie, effect sizes from the same study were not collapsed into a single MES value), and the independent variables we tested are in box 3. The model accounted for the clustering of multiple outcomes from the same study. The regression analysis was performed on three hierarchical databases: training alone (N=55 studies), training with or without supervision (N=73), and training with or without any other intervention components (N=152). We restricted this analysis to studies of professional HCPs, training duration <20 days (studies with missing duration included) and percentage outcomes. Additional details are presented in online supplemental appendix 1 (section 1, pages 3–4).

Box 3 **Variables in the models used to identify in-service training attributes associated with training effectiveness (objective 2)**

**In-service training attributes**

- Training duration (in days).
- Training methods (type and number of methods used): lectures, interactive discussions, clinical practice, role-play and other methods.
- Printed materials for healthcare providers used.
- Computers used for at least part of the training.
- Group size.
- Topic complexity (single vs multiple topics).
- Use of professional trainers (ie, with any training in pedagogical methods).
- Use of trainers who were content experts.
- Some or all training was on-site where healthcare providers routinely work.
- Training delivered over one continuous period versus multiple sessions (eg, a 4-day curriculum delivered via four separate 1-day sessions (eg, four Mondays in a row)).

**Interactions**

- Topic complexity×natural logarithm of training duration.
- Time since training was conducted×whether training was combined with supervision.

**Confounders**

- Baseline performance level.
- Time since training was conducted.

To characterise cost, we analysed group in-service training for professional HCPs, as it was the type tested by the largest number of studies. As studies varied in size, in terms of numbers of HCPs trained and training duration (with longer courses being more expensive), we calculated the cost per HCP per day of training.

All analyses were performed with SAS, V.9.4 (SAS Institute, Inc. Cary, North Carolina, USA).

## Results

### Literature search

The HCPPR screened 216 483 citations and included 2272 reports (online supplemental appendix 1, figure A). Of those, 384 reports were eligible for this analysis. These reports presented 1200 effect sizes from 240 comparisons in 199 studies (see online supplemental appendix 1, tables A1–A4 for sample size details; and online supplemental appendix 2 for study citations and study-specific details). These studies were conducted in 51 LMICs and represented a diversity of methods, geographical settings, HCP types, work environments, health conditions and practices (online supplemental appendix 1, tables B1–B4; and online supplemental appendix 2 for study-specific details). More than half of studies (57.8%) had randomised designs, and 40.2% had a low or moderate risk of bias. Median follow-up time was 4.0 months (from 183 studies that reported follow-up time; IQR: 2.0–7.5), median number of health facilities per study was 16 (from 157 studies; IQR: 6–51) and median number of HCPs per study was 98 (from 125 studies; IQR: 49–167). Most studies (80.4%) were published since 2000. We found no evidence of publication bias (online supplemental appendix 1, figure B).

10.1136/bmjgh-2020-003229.supp2 Supplementary data

### Effectiveness of training strategies (objective 1)

Table 1 presents the effects of training strategies on the practices of professional HCPs. There are five main findings, all supported by low-quality evidence primarily because many studies had a high risk of bias. Since only four study comparisons involved strategies with computers, they were analysed together with studies without computers.

**Table 1:** Effectiveness of training strategies on the practices of professional healthcare providers

Strategies tested*		Outcome scale	No of study comparisons (risk of bias: low, moderate, high, very high)	Median MES†, in %-points (IQR; range)
Intervention arm	Reference arm			
*Group in-service training*				
Group in-service training	Controls	Percentage	60 (9, 19, 17, 15)	7.3‡ (3.6–17.4; –21.3 to 68.1)
Group in-service training	Controls	Continuous	16 (3, 5, 2, 6)	17.4§ (–2.3 to 28.5; –25.0 to 81.4)
Group in-service training plus other strategy components	Other strategy components	Percentage	13 (6, 3, 4, 0)	3.7¶ (–0.1 to 5.8; –2.7 to 23.6)
Group in-service training plus other strategy components	Other strategy components	Continuous	4 (1, 1, 2, 0)	–7.3 (–20.6 to 3.6; –25.8 to 6.4)
*Educational outreach visits*				
Educational outreach visits	Controls	Percentage	8 (0, 2, 3, 3)	9.9 (4.3–20.6; 2.8–30.9)
Educational outreach visits plus other strategy components	Other strategy components	Percentage	3 (2, 1, 0, 0)	21.5 (NA; 5.4–30.7)
*Group in-service training versus (or combined with) educational outreach visits*				
Group in-service training	Educational outreach visits	Percentage	1 (0, 0, 1, 0)	0.8 (NA; NA)
Group in-service training plus other strategy components	Educational outreach visits plus other strategy components	Percentage	2 (2, 0, 0, 0)	–6.4 (NA; –5.8 to –7.0) (ie, both studies favoured educational outreach visits)
Group in-service training plus educational outreach visits	Controls	Percentage	1 (0, 0, 0, 1)	–2.5 (NA; NA)
*Group in-service training versus (or combined with) self-study in-service training*				
Group in-service training	Self-study in-service training	Percentage	2 (1, 1, 0, 0)	9.3 (NA; 4.6–14.0)
Group in-service training plus other strategy components	Self-study in-service training plus other strategy components	Percentage	2 (0, 0, 1, 1)	2.0 (NA; –1.0 to 5.0)
Group in-service training plus self-study in-service training	Controls	Percentage	1 (0, 0, 1, 0)	24.0 (NA; NA)
*Group preservice training*				
Group preservice training	Controls	Percentage	3 (1, 1, 1, 0)	16.9 (NA; 15.0–46.7)
*Peer-to-peer training*				
Peer-to-peer training	Controls	Percentage	1 (0, 0, 0, 1)	4.0 (NA; NA)
Peer-to-peer training plus group in-service training	Controls	Percentage	3 (0, 0, 0, 3)	8.4 (NA; 1.8–66.2)
Peer-to-peer training plus group in-service training plus other strategy components	Other strategy components	Percentage	1 (0, 0, 1, 0)	25.0 (NA; NA)

*See boxes 1 and 2 for descriptions of the strategies and the comparisons, respectively.

†Effect sizes calculated as the intervention arm improvement minus reference arm improvement.

‡Among studies with a low or moderate risk of bias, median MES=5.1 %-points (IQR: 2.5–14.0; range: –3.0 to 42.8); median MES for high or very high risk of bias studies=9.7 (IQR: 5.1–19.8; range: –21.3 to 68.1).

§Among studies with a low or moderate risk of bias, median MES=15.1 %-points (IQR: –3.8 to 21.2; range: –25.0 to 81.4); median MES for high or very high risk of bias studies=20.3 (IQR: 1.9–41.4; range: –19.2 to 57.3).

¶Among studies with a low or moderate risk of bias, median MES=4.5 %-points (IQR: 2.1–5.8; range: –2.0 to 23.6); median MES for high or very high risk of bias studies=1.4 (IQR: –1.8 to 5.4; range: –2.7 to 7.0).

MES, median effect size; NA, not applicable; %-points, percentage-points.

**Figure 1:**
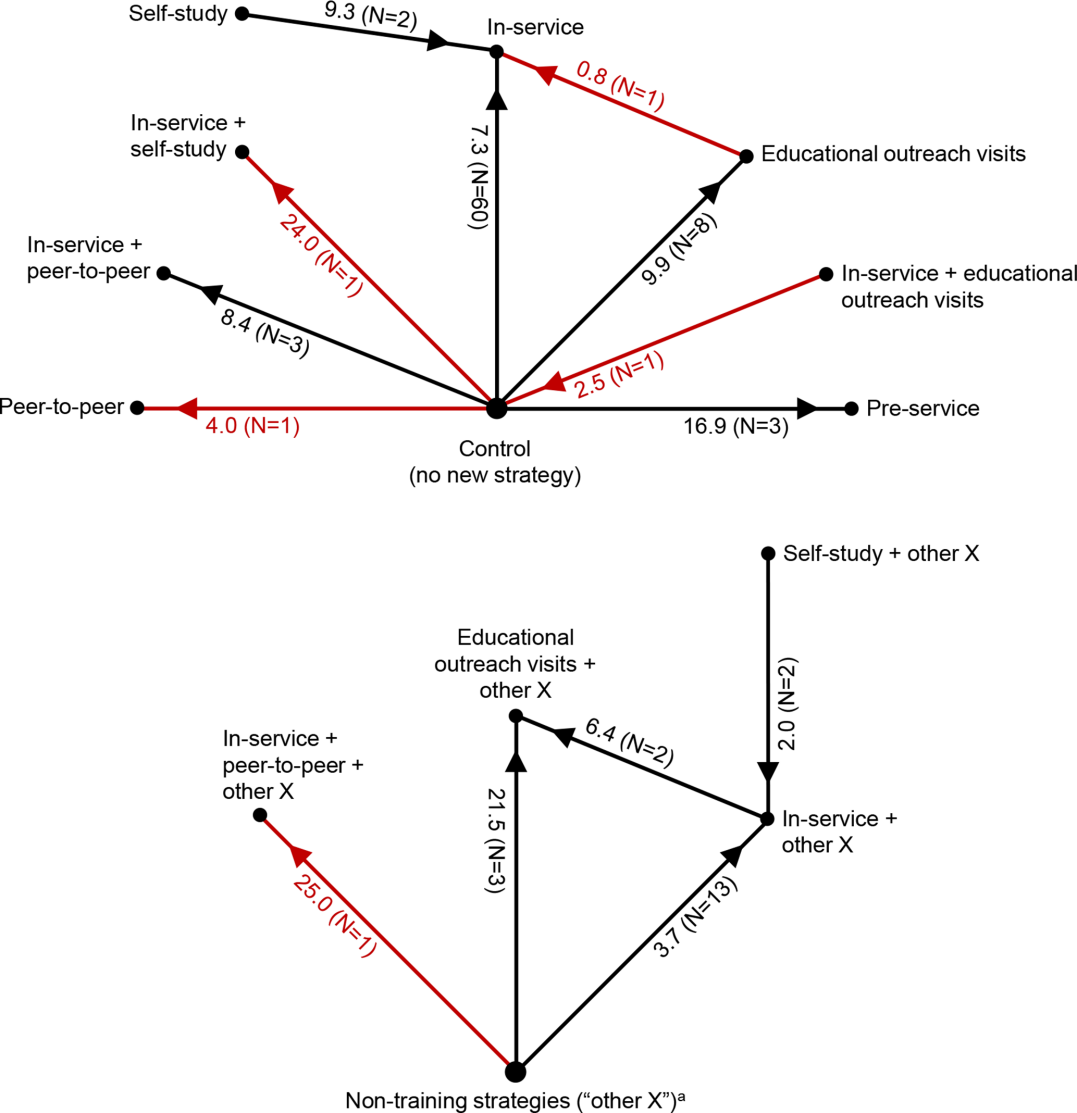
Effectiveness of training strategies for professional healthcare providers in low-income and middle-income countries, as assessed with outcomes expressed as percentages. In-service=group in-service training, pre-service=group preservice training, self-study=self-study in-service training, peer-to-peer=peer-to-peer training, N=number of study comparisons. Red indicates results from a single study, which should be interpreted with caution. The numbers next to each spoke are the median of median effect sizes, in percentage points, and (in parentheses) the number of study comparisons. For each comparison, the arrow points toward the study group with greater effectiveness. For example, preservice training was more effective than controls by a median of 16.9 percentage-points, and (paradoxically) controls were more effective than in-service training plus educational outreach visits by a median of 2.5 percentage-points. ^a^These are non-training strategy components (eg, supervision) that could vary among study comparisons, but are the same for any two arms of a given study comparison, for example, educational outreach visits plus supervision versus supervision.

First, for group in-service training (hereafter referred to as ‘in-service training’), when compared with controls, the median performance improvement ranged from 7.3 to 17.4 %-points (table 1, rows 1–2; figure 1; second and fourth histograms in online supplemental appendix 1, figure D). For example, for a percentage outcome with a typical baseline performance level of 40% and training effect of 7.3 %-points, the post-training performance level would be 47.3%. However, training effects were very heterogeneous: in the largest group of studies (60 comparisons in table 1, row 1), one-quarter of MES values were small (<3.6 %-points) and one-quarter were large (17.4–68.1 %-points). Effect sizes tended to be lower among higher quality studies, with a median improvement of 5.1 %-points for the 28 study comparisons with a low or moderate risk of bias (table 1, footnotes). The one equivalency study of in-service training compared with a gold standard control had a ‘perfect’ effect size of zero (Box 2). The marginal effect of in-service training when added to other strategy components (medians ranging from –7.3 to 3.7 %-points) was smaller than the effect of in-service training when compared with a no-intervention reference group.

Second, educational outreach visits (EOVs) tended to be somewhat more effective than in-service training, although results of individual studies varied widely. The median effect of EOV compared with controls was 9.9 %-points, and the marginal effect when EOV was added to other strategy components was 21.5 %-points (table 1, rows 5–6). Direct comparisons of the two strategies revealed differences that were small (0.8 %-points) to modest (6.4 %-points) (table 1, rows 7–8). The one study (with a very high risk of bias) that combined the two strategies had no effect (–2.5 %-points).

Third, group in-service training might be more effective than self-study by 2.0–9.3 %-points (table 1, rows 10–11). The one study (high risk of bias) that combined both strategies had a large effect (24.0 %-points). Fourth, group preservice training for a small portion (eg, a one-semester course) of a preservice training programme had a moderate effect (16.9 %-points) compared with typical preservice training (table 1, row 13). Fifth, while all studies of peer-to-peer training had a high risk of bias, one study found a small effect of peer-to-peer training (4.0 %-points) (table 1, row 14); and median effects of peer-to-peer training combined with in-service training ranged from 8.4 to 25.0 %-points.

We found only five eligible studies (all high risk of bias) of training strategies to improve lay HCP practices (online supplemental appendix 1, table C). All findings were supported by low-quality evidence. There was essentially no effect for in-service training (median MES=–0.9 %-points) and EOV (0.2 %-points). The effect of group preservice training (one study of a 3-day preservice training programme) was 9.1 %-points.

### Attributes associated with in-service training effectiveness (objective 2)

Modelling of the ‘training alone’ database included 58 comparisons from 55 studies. This database was derived from the 60 ‘group in-service training versus control’ comparisons (table 1, row 1), with two comparisons excluded because training durations were over 20 days (durations unfeasible for most programmes). See online supplemental appendix 1, tables A5–A6 for sample sizes and risk-of-bias categories for all three modelling databases. Adjusted R^2^ values of the models ranged from 0.102 to 0.340, indicating that they explain only a small-to-moderate amount of the variation in effect sizes.

The analyses of attributes associated with group in-service training effectiveness were supported by low-quality evidence primarily because many studies had a high risk of bias. Several attributes had statistically significant associations with training effectiveness (table 2, rows 1–4). The mean effect of in-service training when some or all training was done at the site where HCPs routinely work was 6.0–10.4 %-points greater than when all training was done off-site. In-service training that incorporated clinical practice tended to be more effective than training without this method by 6.9–7.4 %-points. The mean effect of in-service training alone declined with time since training by 0.8–1.0 %-points per month, with training effectiveness waning to zero after 19.8–22.5 months, on average. When training was combined with supervision, the mean effect did not appear to decrease over time (online supplemental appendix 1, figure C), although this result was sensitive to outliers. Finally, lower baseline performance was associated with greater response to training; for every 10 %-point decrease in baseline performance level, mean in-service training effectiveness was 1.1–1.5 %-points higher.

**Table 2:** Associations of training attributes on training effectiveness for the practices of professional healthcare providers (HCPs)

Attribute	Findings
Attributes associated with training effectiveness based on >1 study	
Location of training activities: where HCPs routinely work (on-site) versus all training off-site	Direct evidence*: none. No head-to-head study examined this attribute. Indirect evidence*: having some or all training on-site was more effective than all training off-site by a mean of 6.0–10.4 %-points. Details in online supplemental appendix 1, table E, row 1.
Use of clinical practice as a training method	Direct evidence*: none. No head-to-head study examined this attribute. Indirect evidence*: training with clinical practice was more effective than training without clinical practice by a mean of 6.9–7.4 %-points. Details in online supplemental appendix 1, table E, row 2.
Time since training	Direct evidence*: change over time in the marginal effect of supervision given training was 0.3 %-points per month (p=0.58) for 0.5–5.5 months after training. Indirect evidence*: mean effect of training only (without supervision) decreased by 0.8–1.0 %-points per month after training, with the effect predicted to reach zero after 19.8–22.5 months, on average. Mean effect of training plus supervision did not decrease over time (there was a trend of increasing effect of 0.2–0.3 %-points per month, which was not statistically significant). The latter result was sensitive to outliers. Details in online supplemental appendix 1, table E, row 3, and figure C.
Baseline performance level	Direct evidence*: none. No head-to-head study examined this attribute. Indirect evidence*: mean effect of training decreased by 0.11–0.15 %-points for every 1 %-point increase in baseline performance level. Details in online supplemental appendix 1, table E, row 4.
Attributes associated with training effectiveness based on only 1 study (ie, interpret with caution)	
Tailoring in-service training to HCPs’ stage of readiness to change	Direct evidence*: training tailored to HCPs’ stage of readiness to change was more effective than non-tailored training by a median of 23.3 %-points. Indirect evidence*: none. The HCPPR database did not include this attribute. Details in online supplemental appendix 1, table E, row 5.
Preservice training with group feedback about pretraining evaluation results	Direct evidence*: preservice training with group feedback about pretraining evaluation results was more effective than with individual feedback by 19.0 %-points. Indirect evidence*: none. Modelling was not performed for preservice because there were too few studies. Details in online supplemental appendix 1, table F.
Training on a protocol versus training on clinical acumen	Direct evidence*: training on a protocol-based model (HCPs applied screening results to an algorithm), combined with supervision and integration of services, was more effective than training on clinical acumen (what HCPs did with screening results was left to their discretion), combined with supervision and integration of services, by 8.4 %-points. Indirect evidence*: none. The HCPPR database did not include this attribute. Details in online supplemental appendix 1, table E, row 6.
Attributes with an unclear association with training effectiveness because direct and indirect evidence was contradictory	
Trainee group size	Direct evidence*: small group training (ie, 2–14 participants) was somewhat more effective than large group training (ie, >14 participants), by a median of 5.3 %-points. Indirect evidence*: large group training was somewhat more effective than small group training by a mean of 5.8–6.1 %-points. Details in online supplemental appendix 1, table E, row 11.
Trainers with content expertise	Direct evidence*: training by trainers with content expertise (doctors) was slightly more effective than training by trainers without content expertise (paramedics), by 2.5 %-points. Indirect evidence*: training when ‘all trainers were content experts’ was less effective than when not all trainers were content experts, by a mean of 16.1 %-points. Details in online supplemental appendix 1, table E, row 12.
Use of non-interactive lectures as a training method	Direct evidence*: training with a non-interactive lecture or session (as a sole training method) was more effective than interactive training (as a sole training method) by a median of 5.0 %-points. Indirect evidence*: no significant association. However, all univariable regression coefficient β values were less than 5.0 %-points (range: −2.5 to 3.8 %-points; all non-significant). Details in online supplemental appendix 1, table E, row 13.
Interaction between in-service training duration and the topic complexity of the training	Direct evidence*: for training on single topics, training effectiveness might have increased with course duration; and for training on multiple topics, training effectiveness seemed unrelated to course duration. Indirect evidence*: for training on single topics, training effectiveness was unrelated to course duration; but for training on multiple topics, effectiveness increased with longer course duration. Details in online supplemental appendix 1, table E, row 14.

*Direct evidence comes from head-to-head studies (ie, the direct comparison of two training approaches), and indirect evidence comes from modelling results (ie, essentially the difference between the mean effect of a group of ‘intervention vs control’ comparisons from studies that used one training approach, and the mean effect of a group of ‘intervention vs control’ comparisons with a different training approach).

HCPPR, Health Care Provider Performance Review; %-point, percentage-point.

Several training attributes tested by only one study each had relatively large effects that must be interpreted with caution (table 2, rows 5–7): in-service training tailored to HCPs’ stage of readiness to change (23.3 %-points), preservice training with group feedback (19.0 %-points) and in-service training on a protocol (8.4 %-points). In contrast, several attributes had little or no effect on training effectiveness (<4.5 %-points): training duration; training with computers, interactive methods (eg, role-play) or written materials; training over multiple sessions; number of educational methods employed; training via live video interactive sessions; use of trainers with pedagogical training and topic complexity (online supplemental appendix 1, table E, rows 7–10). The effects of training group size, using trainers with content expertise, non-interactive lectures, and an interaction between training duration and topic complexity were unclear because of conflicting results (table 2, rows 8–11).

### Cost of in-service training

Among 84 study arms of professional HCPs exposed to in-service training (from 68 studies), data on cost or from an economic evaluation of any type were available for only 26 arms or 31.0% (even after actively querying investigators); and 15 arms (from 11 studies) had data that allowed us to calculate cost per HCP per day of training. These 11 studies were from countries in Africa, Asia, Latin America and Europe. The median training duration was 5 days (IQR: 4–9, range: 1–19). The median cost per HCP per day of training was $26 (IQR: 4–72, range: 1–94). Cost was not related to study year (which ranged from 1991 to 2013) or whether the training was done on-site versus off-site. Costs tended to be higher in low-income countries (median = $54/HCP/day, N=5 arms) than in middle-income countries (median = $27/HCP/day, N=10 arms).

## Discussion

In LMICs, training is often used to improve HCP performance and reduce the enormous burden caused by poor quality healthcare. While experts have voiced the need to go beyond training and implement other strategies and health systems interventions,^1–3 15 16^ training will continue to be a core element of most improvement approaches. This analysis of HCPPR data characterises the effectiveness of different training strategies and identifies attributes associated with training effectiveness. Strengths of this analysis are the large number of studies that are all from LMICs, the inclusion of head-to-head studies that directly compare training strategies and quantitative results.

For professional HCPs, certain training strategies appear to be more effective at improving HCP practices: on average, EOV was somewhat more effective than in-service training, while the latter might in turn be more effective than peer-to-peer training or self-study. Certain combinations (eg, in-service training plus self-study) might be more effective than others (eg, in-service training plus EOV). We also identified attributes of in-service training that were associated with larger, sustained improvements: on-site training, incorporating clinical practice and combining training with supervision. While our results suggest that certain approaches are more effective, the variability of results and the overall low-quality of evidence suggest that (as the larger HCPPR emphasised) programmes should monitor performance to understand the effect of a given approach in their specific context.^1^ For the small number of studies of lay HCPs, in-service training had little or no measurable effect. Much more work is needed in this crucial area given the reliance on this cadre of HCPs in many settings.

Despite analysing 199 studies, the overall quality of evidence about training interventions is weak and substantial knowledge gaps remain. An evidence-based research agenda derived from this study (box 4) suggests greater attention to replication of key results, additional studies of specific promising strategies, better reporting on context, and use of more rigorous study designs and standardised methods.

Box 4 **Evidence-based recommendations for strengthening research on training strategies to improve healthcare provider practices in low-income and middle-income countries**

**Regarding topic areas, future research should focus on:**

- Replicating studies of promising strategies tested with only one or a small number of studies.
- Head-to-head comparisons of key training strategies (eg, in-service training vs educational outreach visits), strategy combinations (eg, in-service training plus peer-to-peer training vs in-service training alone) and training attributes (eg, different training methods and durations).
- Use of computers as a training adjunct, especially self-study in-service training with computers, given the increased availability of personal computers and internet-based training courses.
- Rigorous studies of training strategies to improve the practices of lay or community health workers.
- Better quantitative and qualitative understanding of how context influences strategy effectiveness.

**Regarding methods, future research should:**

- Use standardised methods, especially for outcomes, strategy description, implementation (including dose and fidelity) and characterisation of study context.
- Prioritise head-to-head studies, which provide stronger evidence for comparing different training approaches.
- Have rigorous study designs, such as interrupted time series with a randomised comparison group, which reduce bias and show how effectiveness changes over time.
- Prioritise the use of practice outcomes expressed as a percentage (rather than continuous outcomes, which can be more difficult to interpret; see online supplemental appendix 1, figure D).
- Have follow-up periods that match the timeframe that programs require for improvements to be sustained (eg, at least 12 months) and include multiple measures of effect so changes (reductions or further improvements) in effectiveness over time can be quantified.
- Include assessments of strategy cost and cost-effectiveness.
- Be designed to better contribute to filling gaps in the evidence base about strategy choice and combinations of components.*

*Studies directly comparing two training approaches without other components are the easiest to interpret. However, given the generally moderate effect of training as a sole strategy, studies should include other enhancing components in both study arms (eg, training approach A+quarterly supervision vs training approach B+quarterly supervision).

Our estimates of strategy effectiveness are generally similar to those from other reviews, although methodological differences limit comparisons. The review by Forsetlund *et al* (with 11 of 81 studies from LMICs) found the median effect of educational meetings on HCP practices was 6 %-points for dichotomous outcomes and 10 %-points for continuous outcomes.^9^ The effect of provider education reported by Holloway *et al* (all studies from LMICs) was 6 %-points.^17^ A review on educating lay HCPs identified only two studies, which had divergent results: no effect and 22 %-points.^18^ A review on EOV (with 3 of 69 studies from LMICs) found a median effect of 5.6 %-points for dichotomous outcomes.^11^ Notably, an HCPPR-based study of strategy effectiveness over time found a decline in the effect of training plus supervision (0.4–0.5 %-points/month)—the opposite of our result.^19^ As that study analysed effectiveness at multiple follow-up time points per study, its results probably have greater validity.

Certain reviews present lists of recommended attributes for effective training or learning. Some of these attributes agree with our results (eg, on-site training (personal communication from Alison Trump (presentation: ‘Using evidence to design effective learning to support health worker performance’), Jhpiego, 22 August 2019) and Bluestone *et al*)^20^ and combining training with supervision,^8^ while some do not (eg, training over multiple sessions^20^ and interactive methods (personal communication from Alison Trump (presentation: ‘Using evidence to design effective learning to support health worker performance’), Jhpiego, 22 August 2019)), or reflect attributes that we were unable to examine (eg, higher attendance at educational meetings).^9^

Our analysis had important limitations. Included studies had heterogeneous methods and contexts, and many studies had a high risk of bias and short follow-up period. Most training strategies were tested by few studies. Not all potentially relevant training attributes were abstracted (eg, online supplemental appendix 1, box A), and one key attribute (whether training was on-site) had many missing values. The modelling performed on the ‘training with or without other strategy components’ database used a simplistic approach to adjust for the effect of non-training components, which was unlikely to remove all confounding. The lack of association for certain training attributes might be explained by unmeasured factors. For example, training duration might not have been related to effectiveness because courses covered different amounts of material and investigators chose appropriate durations for the content. We did not adjust for multiple comparisons in the modelling, so the results reflect hypothesis screening rather than true hypothesis testing. As results for the attributes associated with training effectiveness come from different models and databases, the results are not necessarily additive. For example, the effectiveness of training that was both on-site (6–10 %-point effect) and used clinical practice (7 %-point effect) might not be 13–17 %-points greater than training without these attributes. The cost results should be interpreted with caution because we had insufficient detail to properly account for inflation and real exchange rates, and because the generalisability of cost data from research projects to implications for programmes across such diverse settings is unclear.

## Conclusions

This analysis has characterised the effectiveness of training strategies and identified attributes associated with training effectiveness in LMICs. Although more higher quality research is needed, for now, decision-makers should consider these results to make HCP training in LMICs more effective and ultimately to strengthen health programmes that serve billions of people.
