# Supplementary material for: The effectiveness of training strategies to improve healthcare provider practices in low-income and middle-income countries
Source: BMJ Glob Health. 2021 Jan 15;6(1):e003229. doi: 10.1136/bmjgh-2020-003229 (PMC7813291; doi:10.1136/bmjgh-2020-003229)
Supplement: Supplementary data [file bmjgh-2020-003229supp001.pdf]

Appendix 1 for:

Rowe AK, Rowe SY, Peters DH, Holloway KA, Ross-Degnan D. The effectiveness of training strategies to improve health care provider practices in low- and middle-income countries

Section 1. Methodological details

Section 2. Additional results

Section 1. Methodological details

Objective 1: Characterize the effectiveness of different training strategies

- To define the non-training part of a strategy (i.e. the "other" strategy components), we used strategy definitions at the "component category" level, rather than the "individual component" level—although the non-training parts of the strategy in two arms from the same study were usually identical at the individual component level. For example, if arm 1 was "training + provision of drug supply" and arm 2 was "provision of drug supply", then the non-training part is the same.

- Several component categories (e.g., printed information for health care providers [HCPs], patient support, and community support) contain numerous individual components. On a few occasions, the individual strategy components between study arms did not exactly match; however, the categories of strategy components matched. For example, in one study comparison: arm 1 had "poster for HCP + poster for community + drug supply" and arm 2 had "training + educational video for community + poster for HCP + poster for community + drug supply." Even though the individual "community support" components were not exactly the same between the arms (Arm 1: poster for community vs. Arm 2: educational video + poster for community), both arms had components in the "community support" category so that the "other" non-training parts of the strategies were the same in both arms.

- If study arm 1 had printed information for HCPs but no training, and study arm 2 had training with printed information for HCPs handed out (originally coded as "training only"), the strategy in arm 2 was re-defined as "training + printed information for HCPs" so that both arms had the same "other" non-training component of "printed information for HCPs". This approach better reflects what each study arm was exposed to and improves the interpretability of the effect sizes.

- Indirect analysis (true control comparisons) and direct analysis (head-to-head comparisons) results were presented together in a "network" diagram (Figure 2). However, network meta-analysis was not performed because it would not have added much to the simpler analysis used. Specifically (as can be seen in Figure 2): in the main network (with the no-intervention control reference group), there is only 1 closed loop (which adds a single study comparison, while the other spokes have 59 and 8 comparisons). Additionally, in the smaller network (with non-training strategy components as the reference group), the "other X" nodes represent a diversity of strategies, so they are not combinable (i.e., they only make sense for "within study" comparisons, which quantify the marginal effect of the training strategy conditioned on other components in the strategy).

Objective 2: Identify attributes of in-service training associated with training effectiveness

- Indirect analysis (modeling of study comparisons with a no-intervention control group)

1. For each of the three databases of training studies (i.e., training only, training +/- supervision, training +/- other strategy components), we created a mixed model with a random-intercept (in which the cluster was the study) using a 4-step approach: 1) univariable analyses of individual training attributes; 2) attributes with univariable p-values < 0.10 were identified; 3) step 2 attributes were included in a multivariable model (except the variable for "some or all training on-site", despite its having a univariable $p < 0.10$, because this variable was missing for about one-fifth of effect sizes*); and 4) if the step 3 model included the "duration-topic complexity" interaction or the "supervision-time" interaction, and that interaction was not significant ($p < 0.05$), then the interaction and components (duration & complexity, or time) were removed from the model. To examine the association for the "some or all training on-site" variable (which was often missing), we developed an alternative model with the following 4-step approach: 1) univariable analyses of individual training attributes; 2) attributes with univariable p-values < 0.10 were identified; 3) step 2 attributes were included in a multivariable model that included the "some or all training on-site" variable; and 4) if the step 3 model included the "duration-topic complexity" interaction or the "supervision-time" interaction, and that interaction was not significant ($p < 0.05$), then the interaction and components (duration & complexity, or time) were removed from the model. For the models containing the "some or all training on-site" variable, we did not consider results for other training attributes (e.g., training duration); these models were only used to evaluate the effect of some/all on-site training (adjusted for other factors, as potential confounders).

2. The following training attributes were excluded because they were highly unbalanced (i.e., one level of the attribute had <5 comparisons): whether training used all four key educational methods (clinical practice, interactive session, non-interactive lecture, and role play), and whether training used computer-based methods.

3. Attempts to add training attributes not included in the step 4 model, or to include non-training effect modifiers, resulted in unstable models. Out of concerns that more complex models might be over-specified, we only tested one set of additional models that included variables for baseline performance and time since training, as they were known predictors of effect size. Thus, for each of the three databases, we had four final models (see Tables D1–D3).
    a) Model 1: no predictors forced into the model
    b) Model 2: baseline performance and time since training forced into the model
    c) Model 3: some/all on-site training forced into the model
    d) Model 4: some/all on-site training, baseline performance, and time since training forced into the model

4. Details on eligibility
    a) Inclusion criteria: 1) professional HCP studies (i.e., no lay HCP predominant studies) with at least one comparison of in-service group training versus a true control, and 2) training duration < 20 days (studies with missing training duration were included).
    b) Exclusion criteria: 1) educational outreach visits, 2) peer-to-peer training, 3) self-study, 4) pre-service training, and 5) equivalency studies.

c) Note. Regarding distance learning, studies were eligible if there was a classroom of trainees with an off-site trainer (i.e., studies of HCPs studying in isolation were considered "self-study" and excluded).

5. Additional details on modeling for the three training databases
   a) Database of studies on training only (see Table D1). See methods above; no additional details.
   b) Database of studies on training +/- supervision (see Table D2). All models included 1 indicator variable for the presence of any supervision.
   c) Database of studies on training +/- other strategy components (see Table D3). All models included nine indicator variables for the presence of non-training components (i.e., community supports, patient supports, strengthen infrastructure, health systems financing and other incentives, governance or regulation, group problem solving, supervision, other management techniques, and information and communication technology for HCPs). Two indicator variables for the presence of other non-training component categories (i.e., HCP-directed financial incentives, and printed information or job aids for HCPs) were excluded from models because they were highly unbalanced (i.e., one level of the variable had <10 comparisons).

* The variable for "some or all training on-site" had missing values for: 93 (25.0%) of 372 observations in the training only dataset, 113 (24.0%) of 470 observations in the training +/- supervision dataset, and 160 (18.7%) of 856 observations in the training +/- other components dataset.

- Direct analysis (head-to head comparisons)

1. Eligible comparisons were: a) in-service training approach A vs. in-service training approach B (e.g., a 6-day training versus an 11-day training), and b) in-service training approach A + other strategy components vs. in-service training approach B + other strategy components.

Box A. Attributes of successful training according to a specialist in the science of how people learn[a]

1. The training uses analogies as bridges to link new knowledge to prior knowledge
2. Trainers actively attempt to identify and address misconceptions directly
3. Information is categorized (i.e., presented using relevant categories)
4. Information is sequenced (i.e., presented in a logical sequence)
5. Trainees practice both individual skills (e.g., performing a skin pinch to evaluate dehydration in a child with diarrhea) <u>and</u> the entire set of desired practices (e.g., all aspects of evaluating a child with diarrhea) (this agrees with Malcolm Knowles Principle of Andragogy #2: Experience should be at the root of all learning tasks and activities)
6. Trainers provide informational feedback (i.e., rather than only praise or criticism)
7. Complex information is simplified
8. Training uses images
9. Training uses mnemonics
10. Training uses stories, case studies, problem-based learning, or simulations (this agrees with Malcolm Knowles Principle of Andragogy #4: Adult learning should be problem-centered, rather than content-oriented)
11. Procedures are broken down into steps
12. The training has breaks to avoid overwhelming trainees
13. Trainees are asked to discuss, debate, or persuade each other
14. Some aspect of the training involves trainees collaborating with each other
15. Trainees are asked to teach each other
16. Trainers help trainees tie the training's objective to a self-relevant, self-transcendent purpose (e.g., for training on treating an illness, trainers helped trainees understand that improving treatment practices will both make them a better, more respected health worker and save the lives of people in their community) (this agrees with Malcolm Knowles Principle of Andragogy #3: Adult learning should have immediate relevance to real life)
17. Trainers recognize trainees growing competence
18. Trainers help trainees develop self-efficacy (i.e., confidence that trainees can perform the required tasks)
19. Trainers ask trainees to make a plan on how the new knowledge would be put to use. The plan includes specifying outcomes and how the outcomes will be measured, and setting goals that are short-terms, specific, and moderately challenging.
20. Trainers should get feedback from adult learners (based on Malcolm Knowles Principle of Andragogy #1: Adult learners must be involved in the design and development of their learning experience).

[a] Adapted from a presentation, citation: *Annie Murphy Paul. "Learning Science." Presentation at the Teach to Reach Summit, Seattle, Washington, November 2, 2015.*

Section 2. Additional results

Section 2a. Detailed flowchart of the literature search of the systematic review on which this study is based

Figure A. Detailed flowchart of the literature search

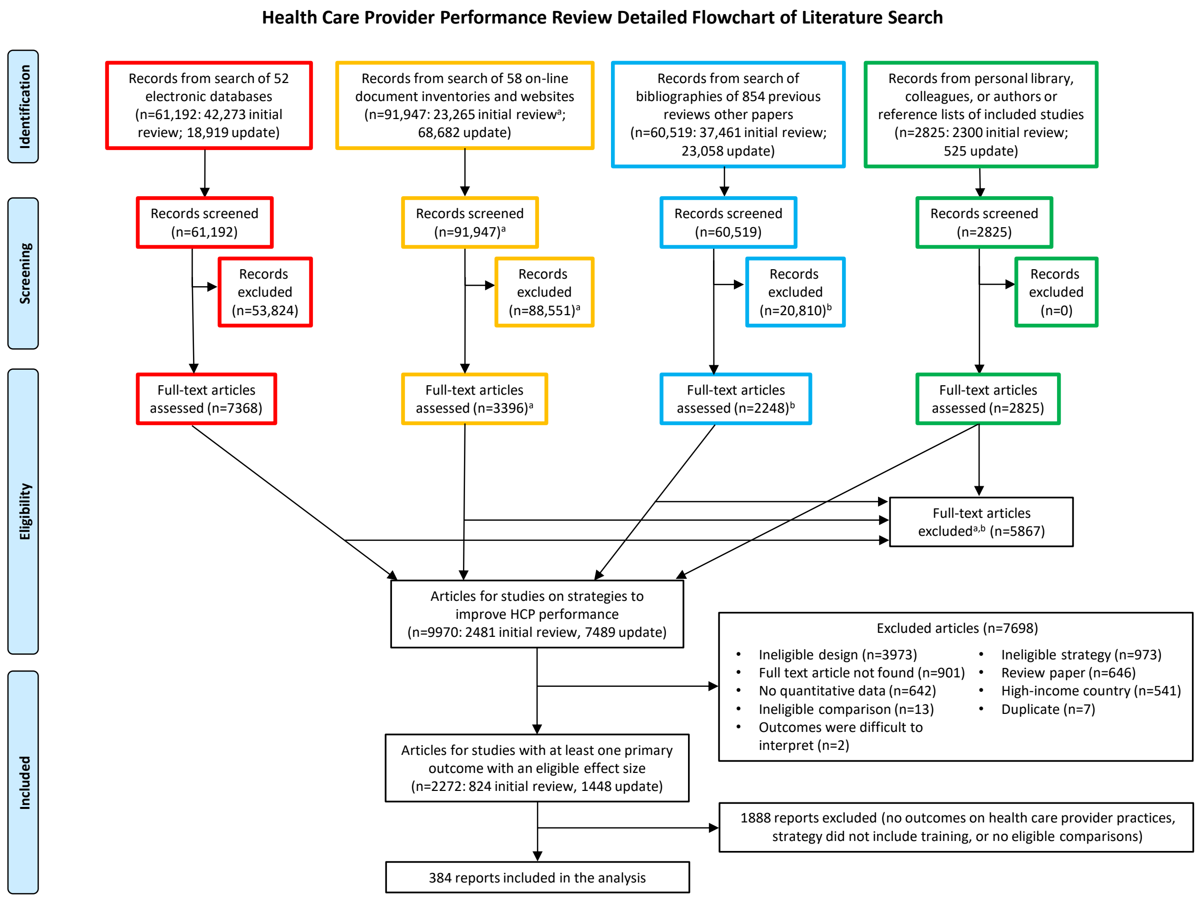

Abbreviation: HCP = health care provider.

[a] Early in the initial review's search of on-line document inventories and websites, detailed records were not kept of the number of citations that were screened. Thus, the number of exclusions is unknown; the exact number of records screened is unknown, but was more than 23,265 (which reflects the number once detailed records began to be kept); the exact number of full-text articles assessed is unknown, but was more than 1202 (which reflects the number once detailed records began to be kept); and the exact number of included articles is unknown, but was more than 205 (which reflects the number once detailed records began to be kept).

[b] Early in the initial review's search of the bibliographies of the 510 previous reviews and other papers, detailed records of the search were not kept. Thus, the number of exclusions and full-text assessments are unknown; and the exact number of included articles is unknown, but was more than 247 (which reflects the number once detailed records began to be kept).

Rowe AK, *et al. BMJ Global Health* 2021; 6:e003229. doi: 10.1136/bmjgh-2020-003229

Section 2b. Sample size information

Table A1. Sample size information: analysis of percentage and continuous practice outcomes for study objectives 1 and 2 combined

Health care provider category	True control comparisons	Head-to-head comparisons	Total
**LHW predominant[a]**	15 effect sizes<br>4 comparisons<br>4 studies	2 effect sizes<br>2 comparisons<br>1 study	17 effect sizes<br>6 comparisons<br>5 studies
**Not LHW predominant**	953[b] effect sizes<br>186 comparisons<br>168 studies	230 effect sizes<br>48 comparisons<br>38 studies	1183 effect sizes<br>234 comparisons<br>194 studies
**Total**	968 effect sizes<br>190 comparisons<br>172 studies	232 effect sizes<br>50 comparisons<br>39 studies	1200 effect sizes<br>240 comparisons<br>199 studies

Abbreviation: LHW = lay health worker.

[a] These studies evaluated the effect of a strategy designed to improve LHW practices, even if other types of health workers were providing services in the study setting.

[b] One effect size for a percentage practice outcome from a study of non-LHW-predominant health care providers was an equivalency comparison with a gold standard control group (COMP_IDnew 3640000112: intervention group: in-service training for midwives vs. control group: no in-service training for physicians).

Table A2. Sample size information: analysis of percentage practice outcomes for study objective 1

Health care provider category	True control comparisons	Head-to-head comparisons	Total
**LHW predominant[a]**	15 effect sizes<br>4 comparisons<br>4 studies	2 effect sizes<br>2 comparisons<br>1 study	17 effect sizes<br>6 comparisons<br>5 studies
**Not LHW predominant**	460[b] effect sizes<br>78 comparisons<br>73 studies	82 effect sizes<br>24 comparisons<br>19 studies	542 effect sizes<br>102 comparisons<br>85 studies
**Total**	475 effect sizes<br>82 comparisons<br>77 studies	84 effect sizes<br>27 comparisons<br>21 studies	559 effect sizes<br>108 comparisons<br>90 studies

Abbreviation: LHW = lay health worker.

[a] These studies evaluated the effect of a strategy designed to improve LHW practices, even if other types of health workers were providing services in the study setting.

[b] Eleven effect sizes from 3 true control comparisons of “in-service training alone” from 2 studies involved a training duration longer than 20 days: 5 effect sizes with a 30-day training (COMP_IDnew 6920000112), 5 effect sizes with a 40-day training (COMP_IDnew 6920000113), and 1 effect size (ES_ID 3640000107007) with a 60-day training, which was also an equivalency comparison.

Table A3. Sample size information: analysis of continuous practice outcomes for study objective 1

Health care provider category	True control comparisons	Head-to-head comparisons	Total
**LHW predominant**[a]	0 effect sizes	0 effect sizes	0 effect sizes
**Not LHW predominant**	27 effect sizes<br>4 comparisons<br>4 studies	10 effect sizes<br>16 comparisons<br>16 studies	37 effect sizes<br>20 comparisons<br>18 studies

Abbreviation: LHW = lay health worker.

[a] These studies evaluated the effect of a strategy designed to improve LHW practices, even if other types of health workers were providing services in the study setting.

Table A4. Sample size information: analysis of percentage practice outcomes for study objective 2

Health care provider category	True control comparisons	Head-to-head comparisons	Total
**LHW predominant**[a]	0 effect sizes	0 effect sizes	0 effect sizes
**Not LHW predominant**	856 effect sizes<br>168 comparisons<br>152 studies	138 effect sizes<br>24 comparisons<br>20 studies	994 effect sizes<br>192 comparisons<br>169 studies

Abbreviation: LHW = lay health worker.

[a] These studies evaluated the effect of a strategy designed to improve LHW practices, even if other types of health workers were providing services in the study setting.

Rowe AK, *et al. BMJ Global Health* 2021; 6:e003229. doi: 10.1136/bmjgh-2020-003229

Table A5. Sample size and risk-of-bias information for the three databases used in the modeling analysis to identify attributes associated with in-service training effectiveness: effect size level (study objective 2)

**Database of studies that tested training only: 372 effect sizes from 58 comparisons from 55 studies**		
Risk of bias category	Frequency	Percent
Very high	126	33.9
High	99	26.6
Moderate	111	29.8
Low	36	9.7
**Database of studies that tested training +/- supervision: 470 effect sizes from 79 comparisons from 73 studies**		
Risk of bias category	Frequency	Percent
Very high	130	27.7
High	147	31.3
Moderate	153	32.6
Low	40	8.5
**Database of studies that tested training +/- other components: 856 effect sizes from 168 comparisons from 152 studies**		
Risk of bias category	Frequency	Percent
Very high	245	28.6
High	232	27.1
Moderate	258	30.1
Low	121	14.1

Table A6. Sample size and risk-of-bias information for the three databases used in the modeling analysis to identify attributes associated with in-service training effectiveness: study level (study objective 2)

**Database of studies that tested training only: 55 studies**			
Risk of bias category	Frequency	Percent	
Very high	15	27.3	
High	16	29.1	
Moderate	15	27.3	
Low	9	16.4	
**Database of studies that tested training +/- supervision: 73 studies**			
Risk of bias category	Frequency	Percent	
Very high	19	26.0	
High	24	32.9	
Moderate	19	26.0	
Low	11	15.1	
**Database of studies that tested training +/- other components: 152 studies**			
Risk of bias category	Frequency	Percent	
Very high	48	31.6	
High	43	28.3	
Moderate	34	22.4	
Low	27	17.8	

Section 2c. Descriptive results of included studies

Table B1. General attributes of included studies

Study attribute	All studies (N=199)
Number of study arms	
1	15 (7.5%)
2	157 (78.9%)
3	20 (10.1%)
4	7 (3.5%)
Total number of study arms across all studies	417
Total number of comparisons across all studies	
Strategy vs. true (no intervention) control group	188 (78.3%)
Strategy A vs. Strategy B with no placebo components	48 (20.0%)
Strategy vs. placebo control group	2 (0.8%)
Strategy A vs. Strategy B with $\geq$1 placebo component	2 (0.8%)
Number of effect sizes per study and comparison	
Median number of effect sizes per study (range)	3 (1–134)
Median number of effect sizes per comparison (range)	2 (1–67)
Study designs	
Pre-post study with randomized controls	75 (37.7%)
Pre-post study with non-randomized controls	68 (34.2%)
Post-only study with randomized controls	37 (18.6%)
Interrupted time series with no controls	15 (7.5%)
Interrupted time series with randomized controls	3 (1.5%)
Interrupted time series with non-randomized controls	1 (0.5%)
Economy of country where study was done	
Low income	79 (39.7%)
Lower-middle income	70 (35.2%)
Upper-middle income	48 (24.1%)
Combination of lower-middle and upper-middle income	1 (0.5%)
Combination of lower and middle income	1 (0.5%)
Risk of bias	
Low	36 (18.1%)
Moderate	44 (22.1%)
High	60 (30.1%)
Very high	59 (29.7%)
WHO region where study was conducted	
Africa	70 (35.2%)
Southeast Asia	44 (22.1%)
America	36 (18.1%)

Study attribute	All studies (N=199)
Western Pacific	27 (13.6%)
Eastern Mediterranean	18 (9.1%)
Europe	3 (1.5%)
Africa, America, Southeast Asia, Western Pacific	1 (0.5%)
Year of publication (or date of document for unpublished reports), by decade	
2010 or later (latest year was 2017)[a]	69 (34.7%)
2000–2009	91 (45.7%)
1990–1999	38 (19.1%)
1980–1989	1 (0.5%)
Data collection methods (multiple responses allowed per study)	
Record or chart review	103 (51.8%)
Interview with patient or patient's caretaker	67 (33.7%)
Observation of HCP-patient interaction	43 (21.6%)
Interview with HCP	27 (13.6%)
Simulated client	27 (13.6%)
Questionnaire for HCP (any administration method)	22 (11.1%)
Physical exam of patient by study team	13 (6.5%)
Exam for HCP (e.g., written test for HCP)	8 (4.0%)
Questionnaire for patient or patient's caretaker	6 (3.0%)
Observation of HCP practices not involving real patients	5 (2.5%)
Case scenario	4 (2.0%)
Observation of facility	3 (1.5%)
HCP self-assessment	3 (1.5%)
Interview with administrator	2 (1.0%)
Observation of patient's or patient caretaker's behaviors	2 (1.0%)
Questionnaire for an administrator	1 (0.5%)
Urban vs. rural study setting	
Urban +/- peri-urban areas	72 (36.2%)
Mix of urban and rural areas	46 (23.1%)
Rural areas only	39 (19.6%)
Town +/- rural areas	10 (5.0%)
Peri-urban areas only	4 (2.0%)
Mix of peri-urban and town areas	1 (0.5%)
Unclear or not stated	27 (13.6%)
Data available on strategy cost or other economic evaluation (from either the study reports or responses from investigators)	72 (36.2%)

Abbreviations: HCP = Health care provider, WHO = World Health Organization.

[a] Many reports from 2016 and all from 2017 either were originally identified as unpublished, but were published by the time of the analysis, or were reports that authors or experts provided after the formal literature search had ended.

Table B2. Settings of included studies: places where services were delivered, who owned or operated the service delivery points, and types of health care providers

Study attribute	All studies (N=199)
Places where services were delivered (multiple responses allowed)	
Outpatient health facility	112 (56.3%)
Hospital outpatient department	55 (27.6%)
Hospital inpatient wards	43 (21.6%)
Household or community setting	23 (11.6%)
Pharmacy	17 (8.5%)
Drug shop	14 (7.0%)
Non-hospital health facility inpatient ward	9 (4.5%)
School	5 (2.5%)
Site in transit to hospital or health facility	1 (0.5%)
Other outpatient setting	3 (1.5%)
Who owns or operates the place where services were delivered (multiple responses allowed per study)	
Public or government	142 (71.4%)
Private, for profit	35 (17.6%)
Community	25 (12.6%)
Private, not for profit	18 (9.1%)
Private, profit status unknown or not reported	15 (7.5%)
Other	3 (1.5%)
Unclear or not reported	15 (7.5%)
Type of health care providers (multiple responses allowed per study)	
Physician	112 (56.3%)
Nurse	89 (44.7%)
Midwife	36 (18.1%)
Nurse aide	36 (18.1%)
Pharmacist assistant or non-pharmacist drug vendor	27 (13.6%)
Pharmacist	24 (12.1%)
Paramedic or unspecified non-physician	24 (12.1%)
Lay health worker	23 (11.6%)
Clinical officer	15 (7.5%)
Health educator or information officer	14 (7.0%)
Midwife aide	9 (4.5%)
Student	6 (3.0%)
Laboratorian	4 (2.0%)
Health care provider, type unspecified	11 (5.5%)
Lay health worker was the predominant type of health care provider	5 (2.5%)

Table B3. Health conditions addressed by included studies

Health condition (multiple responses allowed per study)	No. of studies with at least one effect size related to the health condition, among all 199 studies
Multiple (or all) health conditions	51 (25.6%)
Acute respiratory infections	31 (15.6%)
Pregnancy	29 (14.6%)
Malaria	25 (12.6%)
Diarrhea	25 (12.6%)
Reproductive health (not pregnancy related)	17 (8.5%)
HIV/AIDS +/- other sexually transmitted diseases	15 (7.5%)
Newborn health conditions	14 (7.0%)
Malnutrition	13 (6.5%)
Non-communicable diseases not covered by other categories (e.g., asthma)	10 (5.0%)
Infectious diseases not covered by other categories (e.g., appendicitis)	8 (4.0%)
Mental health	7 (3.5%)
Vaccine-preventable illnesses	7 (3.5%)
Sexually transmitted diseases (HIV/AIDS not specifically included)	6 (3.0%)
General medicine use	5 (2.5%)
Tuberculosis	5 (2.5%)
Child health (not covered by other categories, such was well-baby checks)	3 (1.5%)
Heart disease	3 (1.5%)
Infection prevention	3 (1.5%)
Injuries and trauma	2 (1.0%)
Dental health	1 (0.5%)
Hypertension	1 (0.5%)
Non-malaria parasite	1 (0.5%)
Substance abuse	1 (0.5%)

Table B4. Practice outcome categories of all 1200 effect sizes from the included studies

Outcome	HCP practice outcome scale		Totals for percentage and continuous outcomes combined
	Percentage	Continuous	
Assessment	42 studies<br>54 comparisons<br>215 effect sizes	1 study<br>1 comparison<br>1 effect size	42 studies<br>54 comparisons<br>216 effect sizes
Case management[a]	53 studies<br>61 comparisons<br>121 effect sizes	0 studies<br>0 comparisons<br>0 effect sizes	53 studies<br>61 comparisons<br>121 effect sizes
Chemoprophylaxis	4 studies<br>4 comparisons<br>4 effect sizes	0 studies<br>0 comparisons<br>0 effect sizes	4 studies<br>4 comparisons<br>4 effect sizes
Consultation time	0 studies<br>0 comparisons<br>0 effect sizes	2 studies<br>2 comparisons<br>2 effect sizes	2 studies<br>2 comparisons<br>2 effect sizes
Counseling and communication	51 studies<br>58 comparisons<br>273 effect sizes	2 studies<br>2 comparisons<br>6 effect sizes	52 studies<br>59 comparisons<br>279 effect sizes
Diagnosis	15 studies<br>19 comparisons<br>26 effect sizes	0 studies<br>0 comparisons<br>0 effect sizes	15 studies<br>19 comparisons<br>26 effect sizes
Documentation by HCP	13 studies<br>15 comparisons<br>41 effect sizes	0 studies<br>0 comparisons<br>0 effect sizes	13 studies<br>15 comparisons<br>41 effect sizes
Information accessed by HCP	1 study<br>1 comparison<br>5 effect sizes	0 studies<br>0 comparisons<br>0 effect sizes	1 study<br>1 comparison<br>5 effect sizes
Patient dignity	2 studies<br>2 comparisons<br>5 effect sizes	0 studies<br>0 comparisons<br>0 effect sizes	2 studies<br>2 comparisons<br>5 effect sizes
Referral	14 studies<br>17 comparisons<br>30 effect sizes	0 studies<br>0 comparisons<br>0 effect sizes	14 studies<br>17 comparisons<br>30 effect sizes
Treatment	111 studies<br>142 comparisons<br>427 effect sizes	14 studies<br>16 comparisons<br>28 effect sizes	112 studies<br>143 comparisons<br>455 effect sizes
Universal precautions by HCP	6 studies<br>6 comparisons<br>11 effect sizes	0 studies<br>0 comparisons<br>0 effect sizes	6 studies<br>6 comparisons<br>11 effect sizes
Vaccination	5 studies<br>5 comparisons<br>5 effect sizes	0 studies<br>0 comparisons<br>0 effect sizes	5 studies<br>5 comparisons<br>5 effect sizes
Total	197 studies<br>238 comparisons<br>1163 effect sizes	18 studies<br>20 comparisons<br>37 effect sizes	199 studies<br>240 comparisons<br>1200 effect sizes

Abbreviation: HCP = health care provider

[a] Outcomes that include multiple steps of the case-management pathway (e.g., correct diagnosis and treatment).

Rowe AK, *et al. BMJ Global Health* 2021; 6:e003229. doi: 10.1136/bmjgh-2020-003229

Section 2d. Assessment of publication bias

First, we performed a visual inspection of a funnel plot of the 78 study comparisons of a training strategy versus a no-intervention comparison group from studies of professional health care providers (Figure B). The effect size for a single study comparison was the median of effect sizes of all practice outcomes expressed as a percentage. Our interpretation was that asymmetry (a sign of potential publication bias) was possible but not clear. Second, we used the statistical test proposed by Egger to identify asymmetry (Egger *et al*. BMJ 1997; 315: 629–34). We fit the following model using ordinary least squares linear regression: the dependent variable was the standard normal deviate (i.e., the effect size divided by standard error) and the independent variable was the precision (i.e., 1/standard error). Evidence of possible publication bias was defined as a p-value < 0.1 of the model's intercept. We found no evidence of asymmetry (intercept p-value = 0.65).

Figure B. Funnel plot of 78 study comparisons of a training strategy versus a no-intervention comparison group from studies of professional health care providers (results of percentage outcomes)

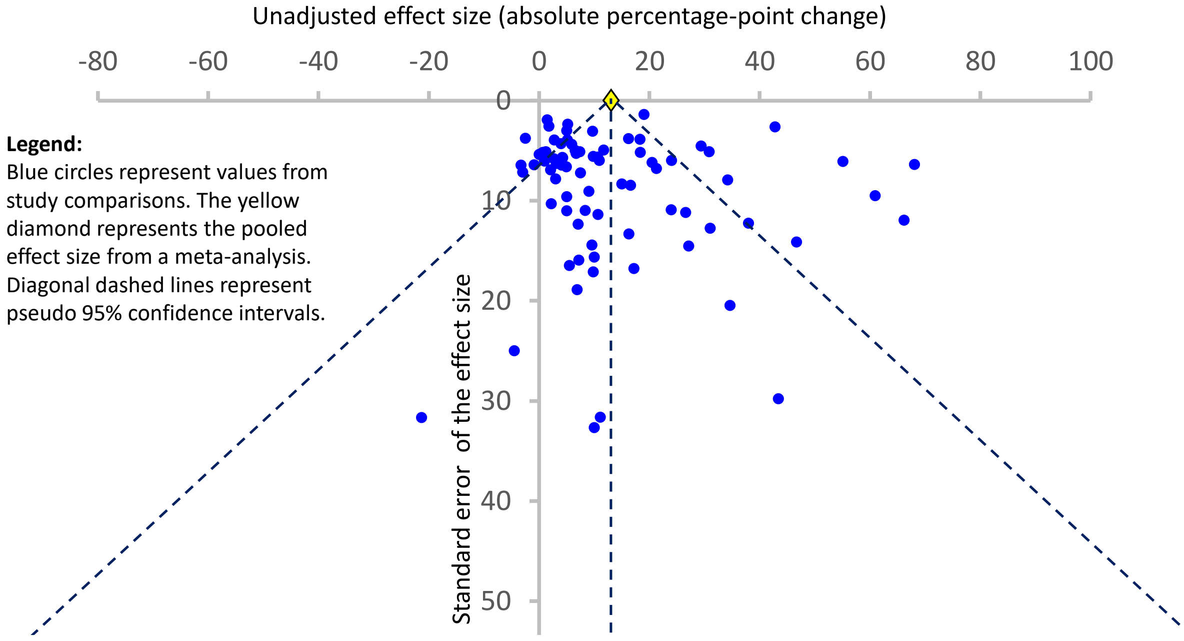

Section 2e. Effectiveness of training strategies

Table C. Effectiveness of training strategies on the practices of lay health care providers

Strategies tested[a]		Outcome scale	No. of study comparisons (risk of bias: low, moderate, high, very high)	Median MES[b] (range)
Intervention arm	Reference arm			
Group in-service training without computers				
Group in-service training without computers	Controls	Percentage	3 (0, 0, 1, 2)	–0.9 (–1.2, 5.6)
Educational outreach visits				
Educational outreach visits plus other strategy components	Other strategy components	Percentage	1 (0, 0, 1, 0)	0.2 (NA)
Group in-service training without computers versus educational outreach visits				
Educational outreach visits	Group in-service training without computers	Percentage	1 (0, 0, 1, 0)	0.7 (NA)
Group pre-service training without computers				
Group pre-service training without computers	Controls	Percentage	1 (0, 0, 0, 1)	9.1 (NA)

Abbreviations: MES = median effect size, NA = not applicable.

[a] See Boxes 1 and 2 in the main article for descriptions of the strategies and the comparisons, respectively.

[b] Effect sizes calculated as the intervention arm improvement minus reference arm improvement.

Rowe AK, *et al. BMJ Global Health* 2021; 6:e003229. doi: 10.1136/bmjgh-2020-003229

Section 2f. Training attributes associated with training effectiveness for professional health care providers

Table D1. Group in-service training attributes associated with training effectiveness: modeling results from studies of training only

Training attribute or other predictor of effectiveness	Model 1: no predictors forced into the model		Model 2: baseline performance and time since training forced into the model		Model 3: on-site training forced into the model		Model 4: on-site training, baseline performance, and time since training forced into the model	
	β	p-value	β	p-value	β	p-value	β	p-value
Intercept	31.0	0.0003	33.1	<0.0001	26.8	0.008	32.2	0.0001
Small training group size (2–14 trainees)	–6.4	0.058	–6.1	0.041	–9.5	0.038	–8.5	0.034
Trainers had content expertise[a]	–16.1	0.041	NA		–15.1	0.099	NA	
Natural logarithm of training duration, in days	NA		–5.3	0.060	NA		–5.2	0.068
Training on multiple topics	NA		–13.4	0.016	NA		–13.9	0.023
Interaction between "logarithm of training duration" and "multiple topics"	NA		11.4	0.006	NA		10.9	0.012
Baseline performance	NA		–0.13	0.027	NA		–0.18[b]	0.013
Time since training, in months	NA		–1.0	<0.0001	NA		–1.0	0.0001
On-site training[c]	NA		NA		10.0	0.031	9.9	0.018
Adjusted R^2	0.110		0.231		0.203		0.340	
No. of observations missing	35/372 (9.4%)		23/372 (6.2%)		107/372 (28.8%)		111/372 (29.8%)	

Abbreviation: NA = Not applicable, which indicates that a predictor was not included in the model, either because the univariable p-value for a training attribute was $\geq$ 0.10 or because the predictor was a potential confounder that was not forced into the model.

[a] For example, a training on managing infectious diseases was taught by a physician with infectious diseases specialist.

[b] Although this result is statistically significant, it was not used because this model was only used to evaluate the effect of on-site training. Results for other factors (e.g., baseline performance) were not considered as valid as those from other models (without the on-site variable) because this model was based on a database with many missing values.

[c] At least some of the training was conducted where the health care provider routinely worked.

Rowe AK, *et al. BMJ Global Health* 2021; 6:e003229. doi: 10.1136/bmjgh-2020-003229

Table D2. Group-in-service training attributes associated with training effectiveness: modeling results from studies of training with or without supervision

Training attribute or other predictor of effectiveness	Model 1: no predictors forced into the model		Model 2: baseline performance and time since training forced into the model		Model 3: on-site training forced into the model		Model 4: on-site training, baseline performance, and time since training forced into the model	
	β	p-value	β	p-value	β	p-value	β	p-value
Intercept	20.8	<0.0001	28.2	<0.0001	17.0	0.0002	25.2	<0.0001
Supervision included in strategy	–5.7	0.053	–5.1	0.078	–4.5	0.15	–2.8	0.40
Small training group size (2–14 trainees)	NA		–5.8	0.035	NA		–8.8	0.013
Training delivered over multiple sessions[a]	–3.8	0.39	NA		–4.0	0.31	NA	
Training duration, in days	–0.6	0.11	–0.8	0.10	–0.4	0.31	–0.5	0.30
Interaction between "supervision" and "time since training"	1.1	0.011	1.0	0.019	0.9	0.036	0.7	0.14
Baseline performance	NA		–0.11	0.025	NA		–0.14[b]	0.017
Time since training, in months	–0.8	0.0003	–0.8	<0.0001	–0.8	0.003	–0.8	0.001
On-site training[c]	NA		NA		7.7	0.059	10.4	0.007
Adjusted R^2	0.102		0.177		0.170		0.280	
No. of observations missing	34/470 (7.2%)		45/470 (9.6%)		125/470 (26.6%)		135/470 (28.7%)	

Abbreviation: NA = Not applicable, which indicates that a predictor was not included in the model, either because the univariable p-value for a training attribute was $\geq$ 0.10 or because the predictor was a potential confounder that was not forced into the model.

[a] For example, a 4-day curriculum delivered via four separate 1-day sessions (e.g., four Mondays in a row).

[b] Although this result is statistically significant, it was not used because this model was only used to evaluate the effect of on-site training. Results for other factors (e.g., baseline performance) were not considered as valid as those from other models (without the on-site variable) because this model was based on a database with many missing values.

[c] At least some of the training was conducted where the health care provider routinely worked.

Rowe AK, *et al. BMJ Global Health* 2021; 6:e003229. doi: 10.1136/bmjgh-2020-003229

Table D3. Group in-service training attributes associated with training effectiveness: modeling results from studies of training with or without other strategy components

Training attribute or other predictor of effectiveness	Model 1: no predictors forced into the model		Model 2: baseline performance and time since training forced into the model		Model 3: on-site training forced into the model		Model 4: on-site training, baseline performance, and time since training forced into the model	
	β	p-value	β	p-value	β	p-value	β	p-value
Intercept	13.7	<0.0001	19.6	<0.0001	11.5	<0.0001	17.9	<0.0001
Community support included in strategy	–0.3	0.94	5.6	0.12	2.2	0.67	7.2	0.12
Patient support included in strategy	–5.4	0.15	–3.2	0.37	–7.6	0.059	–4.5	0.23
Strengthening infrastructure included in strategy	2.6	0.55	3.8	0.38	4.2	0.36	4.5	0.32
Health system financing or other incentives included in strategy	2.7	0.61	2.0	0.73	3.5	0.52	1.4	0.82
Regulation or governance included in strategy	2.8	0.59	–2.7	0.59	1.9	0.71	–1.5	0.80
Group problem solving included in strategy	12.0	0.12	14.8	0.055	10.1	0.19	13.0	0.083
Supervision included in strategy	0.8	0.68	–0.1	0.95	–0.1	0.97	–0.6	0.73
Other management techniques included in strategy	6.6	0.12	7.0	0.078	8.8	0.032	7.5	0.066
Information and communication technology included in strategy	–2.1	0.74	–1.9	0.75	–4.0	0.52	–3.8	0.51
Training included clinical practice for health care providers	6.9	0.013	7.4	0.0068	5.6	0.056	6.4	0.029
Baseline performance	NA		–0.15	0.0001	NA		–0.16[a]	0.0001
Time since training, in months	NA		–0.2	0.14	NA		–0.2	0.27
On-site training[b]	NA		NA		5.0	0.092	6.0	0.045
Adjusted R^2	0.106		0.199		0.153		0.270	
No. of observations missing	0/856 (0%)		59/856 (6.9%)		160/856 (18.7%)		203/856 (23.7%)	

Abbreviation: NA = Not applicable, which indicates that a predictor was not included in the model, either because the univariable p-value for a training attribute was $\geq$ 0.10 or because the predictor was a potential confounder that was not forced into the model.

[a] Although this result is statistically significant, it was not used because this model was only used to evaluate the effect of on-site training. Results for other factors (e.g., baseline performance) were not considered as valid as those from other models (without the on-site variable) because this model was based on a database with many missing values.

[b] At least some of the training was conducted where the health care provider routinely worked.

Table E. Associations of group in-service training attributes on training effectiveness for the practices of professional health care providers: detailed results

Finding	Supporting evidence
Attributes associated with training effectiveness based on > 1 study	
Effect of training when some or all training was done on-site (where HCPs routinely work) was greater than when all training was done off-site by 6.0 to 10.4 %-points	**Direct evidence (results of head-to-head studies)**<br>• None. No head-to-head study examined this attribute.<br><br>**Indirect evidence (model results)**<br>• Some or all training on-site was more effective than all training off-site by a mean of:<br>➢ 9.9 %-points (Table D1, Model 4, row 9: β = 9.9, p = 0.018)<br>➢ 10.0 %-points (Table D1, Model 3, row 9: β = 10.0, p = 0.031)<br>➢ 10.4 %-points (Table D2, Model 4, row 9: β = 10.4, p = 0.007)<br>➢ 6.0 %-points (Table D3, Model 4, row 14: β = 6.0, p = 0.045)
Effect of training that used clinical practice as a training method was greater than training that did not use this method by 6.9 to 7.4 %-points	**Direct evidence (results of head-to-head studies)**<br>• None. No head-to-head study examined this attribute.<br><br>**Indirect evidence (model results)**<br>• Training with clinical practice was more effective than training without clinical practice by a mean of:<br>➢ 6.9 %-points (Table D3, Model 1, row 11: β = 6.9, p = 0.013)<br>➢ 7.4 %-points (Table D3, Model 2, row 11: β = 7.4, p = 0.0068)

Effect of training alone decreased over time since training, but the effect of training combined with supervision did not decrease over time	**Direct evidence (results of head-to-head studies)**<br>• Studies that compared training +/- other components versus training + supervision +/- other components (to estimate the marginal effect of supervision given training +/- other components) with at least two post-intervention measures (to allow an assessment of marginal effect over time) found (based on ordinary least squares linear regression modeling) that the change in the marginal effect of supervision over time was 0.3 %-points per month, although this change was not statistically significant (p = 0.58) (N = 3 MES from 2 studies with a total of 56 post-intervention measures; no. of comparisons with ROB L/M/H/VH: 0/3/0/0, and no. of studies with ROB L/M/H/VH: 0/2/0/0; study follow-up time ranged from 0.5 to 5.5 months). While this analysis does not provide strong support for the interaction (ideally, one would want a marginal effect that significantly increases over time up to at least 20 months after training, which corresponds to the increasing distance between the two lines in Figure C), at least the results match what is predicted by the models of true-control studies (see below) for 0.5 to 5.5 months after training.<br><br>**Indirect evidence (model results)**<br>• Effect of training alone decreased over time since training; but the effect of training combined with supervision did not decrease over time.<br>➢ Table D2, Model 1 (row 6, $\beta_{interaction}$ = 1.1, p = 0.011)<br>➢ Table D2, Model 2 (row 6, $\beta_{interaction}$ = 1.0, p = 0.019)<br>• Effect of training alone (without supervision) decreased over time<br>➢ Table D1, Model 2 (row 8, β = –1.0 %-points per month, p < 0.0001). If one assigns mean values for other variables in the model (baseline performance = 42.7%, ln[training duration] = 1.187, proportion of effect sizes from studies of training with multiple topics = 0.45, and proportion of effect sizes from studies of training with small group size = 0.25), then the training effect size is predicted to reach zero after 19.8 months, on average.<br>➢ Table D2, Model 1 (row 8, β = –0.8 %-points per month, p = 0.0003). If one assigns mean values for other variables in the model (training duration = 4.19 days, proportion of effect sizes from studies of training over multiple sessions = 0.14), then the training effect size is predicted to reach zero after 22.5 months, on average.<br>➢ Table D2, Model 2 (row 8, β = –0.8 %-points per month, p < 0.0001). This regression model is represented by the green line in Figure C. If one assigns mean values for other variables in the model (baseline performance = 40.2%, training duration = 4.0 days, proportion of effect sizes from studies of training with small group size = 0.33), then the training effect size is predicted to reach zero after 22.0 months, on average.<br>• Effect of training combined with supervision did not decrease over time<br>➢ Table D2, Model 1. If one assigns mean values for other variables in the model (training duration = 4.19 days, proportion of effect sizes from studies of training over multiple sessions = 0.14), then: effect size = 12.2 + (0.3 x time). P-value for time = 0.37.<br>➢ Table D2, Model 2. If one assigns mean values for other variables in the model (baseline performance = 40.2%, training duration = 4.0 days, proportion of effect sizes from studies of training with small group size = 0.33), then: effect size = 13.6 + (0.2 x time). P-value for time = 0.64. This regression model is represented by the blue line in Figure C.
Mean effect of training increased by 1.1 to 1.5 %-points for every 10 %-point decrease in baseline performance level	**Direct evidence (results of head-to-head studies)**<br>• None. No head-to-head study examined this attribute.<br><br>**Indirect evidence (model results)**<br>• Mean effect of training decreased as baseline performance level increased<br>➢ From Table D1, Model 2, row 7: β = –0.13 %-points per 1 %-point increase in baseline performance level, p = 0.027.<br>➢ From Table D2, Model 2, row 7: β = –0.11 %-points per 1 %-point increase in baseline performance level, p = 0.025.<br>➢ From Table D3, Model 2, row 12: β = –0.15 %-points per 1 %-point increase in baseline performance level, p = 0.0001.

Attributes associated with training effectiveness based on only 1 study (i.e., interpret with caution)	
Training tailored to HCPs' stage of readiness to change was more effective than non-tailored training by 23.3 %-points	**Direct evidence (results of head-to-head studies)**<br>• Training tailored to HCPs' stage of readiness to change was more effective that non-tailored training by a median of 23.3 %-points (N = 2 MES from 1 study; range: 11.0, 35.5; no. of comparisons with ROB L/M/H/VH: 0/0/2/0, and no. of studies with ROB L/M/H/VH: 0/0/1/0; average study follow-up time: 2 months).<br><br>**Indirect evidence (model results)**<br>• None. No modeling results because the HCPPR database did not include this attribute.
Training on protocol-based model (HCPs applied screening results to an algorithm) was more effective than training on clinical acumen (HCPs used their discretion) by 8.4 %-points	**Direct evidence (results of head-to-head studies)**<br>• Training on a protocol-based model (HCPs applied screening results to an algorithm), combined with supervision and integration of services, was more effective than training on clinical acumen (what HCPs did with screening results was left to their discretion), combined with supervision and integration of services, by 8.4 %-points (N = 1 MES from 1 study; no. of comparisons and studies with ROB L/M/H/VH: 1/0/0/0; average study follow-up time: 11 months).<br><br>**Indirect evidence (model results)**<br>• None. No modeling results because the HCPPR database did not include this attribute.
Attributes with a small or no association with training effectiveness (i.e., magnitude < 5 %-points)	
Effects of training with computers and without computers were similar	**Direct evidence (results of head-to-head studies)**<br>• Training without computers was slightly more effective than training with computers by a median of 0.7 %-points (N = 2 MES from 2 studies; range: -1.2, 2.5; no. of comparisons with ROB L/M/H/VH: 2/0/0/0, and no. of studies with ROB L/M/H/VH: 2/0/0/0; average study follow-up time: 2.5 months).<br><br>**Indirect evidence (model results)**<br>• None. Attribute was not assessed by modeling because of highly unbalanced data.
Training delivered by an in-person trainer was slightly more effective than distance training via live video interactive sessions by 3.6 %-points	**Direct evidence (results of head-to-head studies)**<br>• Training delivered by an in-person trainer was slightly more effective than distance training via live video interactive sessions by 3.6 %-points (N = 1 MES from 1 study; no. of comparisons and studies with ROB L/M/H/VH: 0/1/0/0; study follow-up time: 0.03 months).<br>• Note that "in-person training + in-person supervision" was more effective than "distance learning training + distance supervision", by 5.85 %-points (N = 1 MES). However, this was not a clean comparison of distance versus in-person training because of different supervision approaches.<br><br>**Indirect evidence (model results)**<br>• None. No modeling results because the HCPPR database did not include this attribute.
Effects of training delivered over one session versus multiple sessions were similar	**Direct evidence (results of head-to-head studies)**<br>• Training delivered in one (8 day) session was slightly more effective than 5 days of training over multiple sessions by 0.6 %-points (N = 1 MES from 1 study; no. of comparisons and studies with ROB L/M/H/VH: 0/0/0/1; study follow-up time: 34.5 months [note that, given the likely decay of training effect over time, the long follow-up time of this study greatly limits its utility]).<br><br>**Indirect evidence (model results)**<br>• No significant association. The one univariable β value was -3.8 %-points ($p = 0.39$) (Table D2, Model 1, row 4), which suggests that training over multiple sessions was slightly less effective than training delivered in one session.

Seven other attributes were not significantly associated with training effectiveness	**Direct evidence (results of head-to-head studies)**<br>• None. No head-to-head study examined these attributes, except for duration, which had no clear pattern (see last row of this table).<br><br>**Indirect evidence (model results)**<br>• All the following attributes had univariable p-values > 0.1.<br>➢ Training duration in days (as continuous variable, and coded as > 10 days versus $\leq$ 10 days, and > 13 days versus $\leq$ 13 days)<br>➢ Training used role play<br>➢ No. of educational methods used (continuous, including clinical practice, interactive sessions, non-interactive sessions, role play, and other method)<br>➢ Training used both interactive sessions and non-interactive lectures<br>➢ Training used written materials<br>➢ Trainers with pedagogical training<br>➢ Topic complexity
Attributes with an unclear association with training effectiveness because direct and indirect evidence was contradictory	
Effect of trainee group size is unclear because direct and indirect evidence had contradictory results	**Direct evidence (results of head-to-head studies)**<br>• Small group training (i.e., 2–14 participants) was somewhat more effective than large group training (i.e., > 14 participants) by a median of 5.3 %-points (N = 4 MES from 3 studies; range: –6.5, 18.0; no. of comparisons with ROB L/M/H/VH: 0/0/3/1, and no. of studies with ROB L/M/H/VH: 0/0/2/1; average study follow-up time: 2.7 months)<br><br>**Indirect evidence (results of model results)**<br>• Large group training was somewhat more effective than small group training by a mean of 6.1 %-points (Table D1, Model 2, row 2: β = –6.1, p = 0.041 [note that the reference group was large group size, so the negative β value of –6.1 means that small group was less effective than large group])<br>• Large group training was somewhat more effective than small group training by a mean of 5.8 %-points (Table D2, Model 2, row 3: β = –5.8, p = 0.035 [note that the reference group was large group size, so the negative β of –5.8 means that small group was less effective than large group])
Effect of having trainers with content expertise is unclear because direct and indirect evidence had contradictory results	**Direct evidence (results of head-to-head studies)**<br>• Training by trainers with content expertise (doctors) was slightly better than training by trainers without content expertise (paramedics) by 2.5 %-points (N = 1 MES from 1 study; no. of comparisons with ROB L/M/H/VH: 0/0/1/0, and no. of studies with ROB L/M/H/VH: 0/0/1/0; average study follow-up time: 0.03 months)<br><br>**Indirect evidence (model results)**<br>• Training when "all trainers were content experts" was lower than when not all trainers were content experts by a mean of 16.1 %-points (Table D1, Model 1, row 3: β = –16.1, p = 0.041 [note that the reference group was not having all trainers who were content experts, so the negative β value of –16.1 means that having all trainers who were content experts was less effective than not having all trainers who were content experts])

Effect of training with non-interactive lectures is unclear because direct and indirect evidence had contradictory results	**Direct evidence (results of head-to-head studies)**<br>• Among "training A only versus training B only" study comparisons, training with a non-interactive lecture or session was better than interactive training by 4.3 %-points (N = 1 MES from 1 study; no. of comparisons and studies with ROB L/M/H/VH: 0/0/1/0).<br>• Among "training A only versus training B only" study comparisons and "training A + other strategy components X versus training B + other strategy components X" studies, training with a non-interactive lecture or session was better than interactive training, by median of 5.0 %-points (N = 2 MES from 2 studies; non-interactive minus interactive differences: 4.3 and 5.7 %-points; no. of comparisons and studies with ROB L/M/H/VH: 0/0/2/0).<br><br>**Indirect evidence (model results)**<br>• No significant association. Variable in modeling analysis included any interactive training method (i.e., interactive session, clinical practice, or role play). All univariable β values were less than 5.0 %-points (range: -2.5 to 3.8 %-points; all non-significant, with p-value ranging from 0.15 to 0.95).
Effect of an interaction between the natural logarithm of training duration and topic complexity of the training (single topic versus multiple topics) is unclear because direct and indirect evidence had contradictory results	**Direct evidence (results of head-to-head studies)**<br>• For training on single topics, training effectiveness might have increased with course duration: a 5-day course was more effective than a 3-day course by 8.7 %-points, a 3-day course (plus peer education, supplies, and incentives) was more effective than a 1-day course (plus peer education, supplies, and incentives) by 13.0 %-points, and a 2-day course (plus peer education) was as effective as a 1.5-day course (plus peer education) with a difference of 0.8 %-points.<br>• For training on multiple topics, training effectiveness seemed unrelated to course duration: an 11-day course was as effective as a 6-day course (difference of 0.3 %-points), and an 8-day course was as effective as a 5-day course (difference of 0.6 %-points).<br><br>**Indirect evidence (model results)**<br>• For training on single topics, training effectiveness was unrelated to course duration; but for training on multiple topics, effectiveness increased with longer course duration (Table D1, Model 2, rows 4–6, $\beta_{interaction}$ = 11.4, p = 0.006). If one assigns mean values for other variables in the model (average baseline performance [42.7%], average follow-up time [5.15 months], and proportion of effect sizes from small-group-size trainings [0.252]), then:<br>➢ For training on single health topics: effect size = 20.9 – (5.3 x ln[course duration in days]). P-value for ln[training duration] = 0.06 (Table D1, Model 2, row 4).<br>➢ For training on multiple health topics: effect size = 7.5 + (6.1 x ln[course duration in days]). P-value for ln[training duration] = 0.0475.

Abbreviations: %-points = percentage points, HCP = health care provider, HCPPR = The Health Care Provider Performance Review, MES = median effect size(s), ROB L/M/H/VH = "risk of bias categories: low/moderate/high/very high".

Figure C. The effect of in-service training over time since training, stratified by the presence of supervision, among studies of professional health care providers with training less than 20 days (analysis of the database of training with or without supervision)

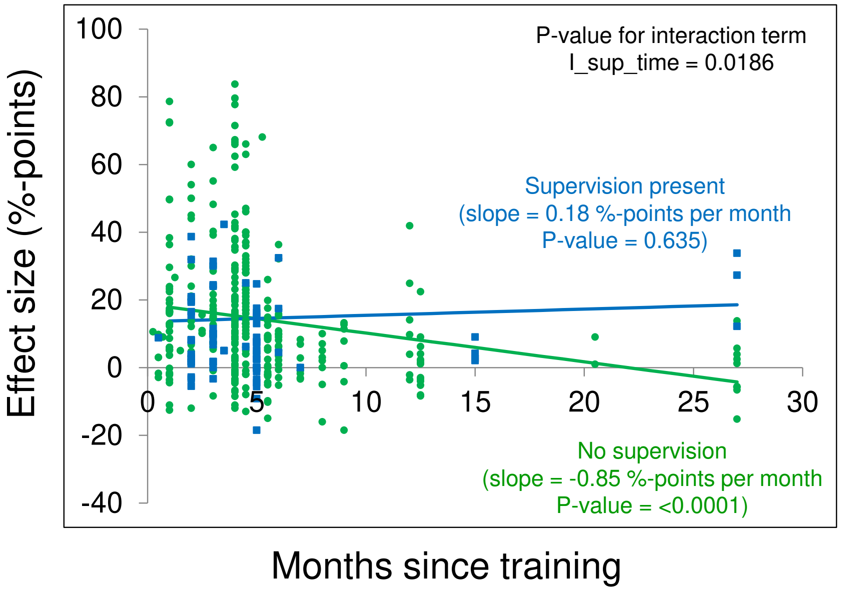

Note 1. Predicted effect sizes assumed average baseline (40.2%), proportion of effect sizes from small-group trainings (0.33), and training duration (4 days).

Note 2. These modeling results were sensitive to the two studies with 27-month follow-up times (one study with supervision and another study without supervision, both with a high risk of bias). When these two studies were removed: a) the time trend for the “no supervision” group was –0.93 %-points per month (p = 0.018), b) the time trend for the “supervision present” group was –0.87 %-points per month (p = 0.055), and c) the interaction term had a value of 0.06 (p = 0.92).

Table F. Associations of pre-service training attributes on training effectiveness for the practices of professional health care providers: detailed results

Finding	Supporting evidence
Attributes associated with training effectiveness based on only 1 study (i.e., interpret with caution)	
Pre-service training with group feedback about pre-training evaluation results was more effective than with individual feedback, by 19.0 %-points	**Direct evidence (results of head-to-head studies)**<br>• Pre-service training with group feedback about pre-training evaluation results was more effective than with individual feedback by 19.0 %-points (N = 1 MES from 1 study; no. of comparisons and studies with ROB L/M/H/VH: 1/0/0/0; study follow-up time: 0.03 months)<br><br>**Indirect evidence (model results)**<br>• None. Modeling was not performed for pre-service because there were too few studies.

Abbreviations: %-points = percentage points, MES = median effect size(s), ROB L/M/H/VH = "risk of bias categories: low/moderate/high/very high".

Figure D. Distributions of effect sizes and median effect sizes for percentage and continuous outcomes for comparisons of group in-service training alone versus controls

Histogram of effect sizes of percentage outcomes

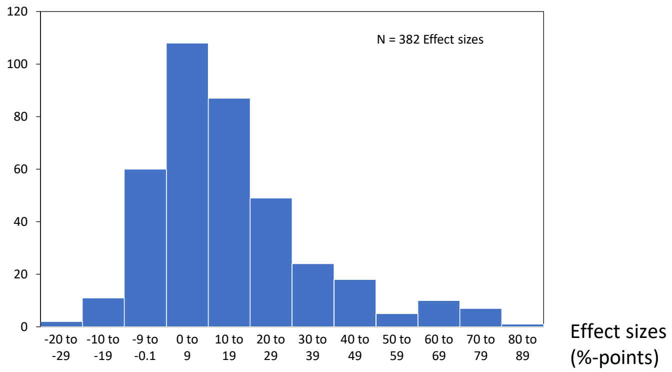

Histogram of median effect sizes of percentage outcomes

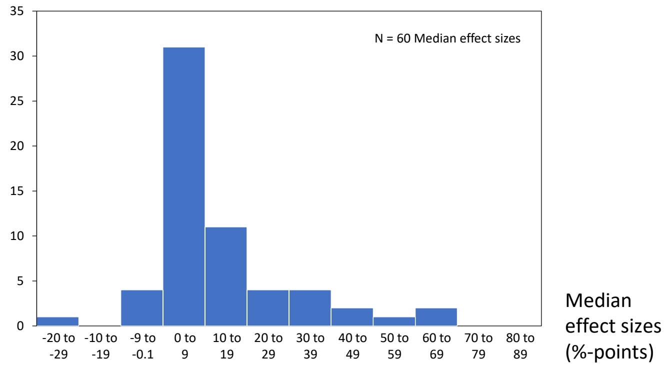

Histogram of effect sizes of continuous outcomes

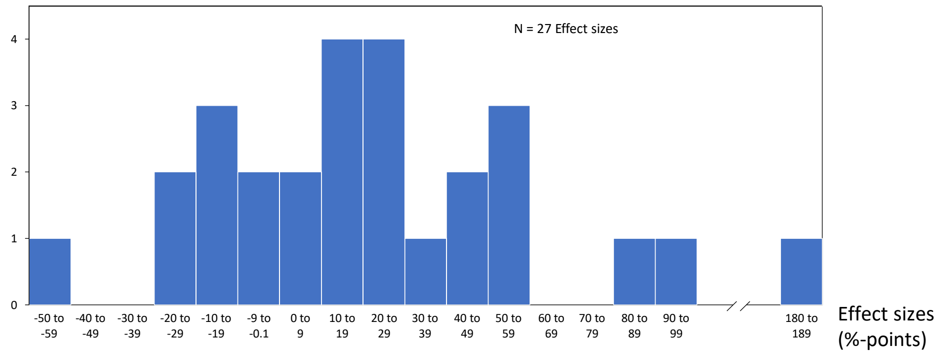

Histogram of median effect sizes of continuous outcomes

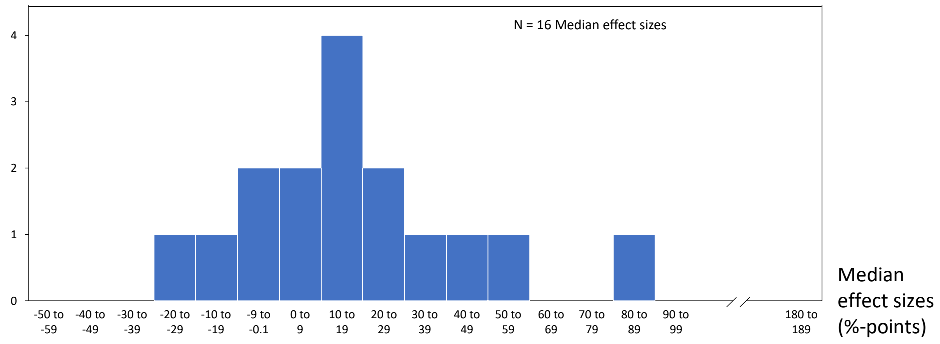

Rowe AK, *et al. BMJ Global Health* 2021; 6:e003229. doi: 10.1136/bmjgh-2020-003229
